# Soil Bacterial and Archaeal Communities and Their Potential to Perform N-Cycling Processes in Soils of Boreal Forests Growing on Well-Drained Peat

**DOI:** 10.3389/fmicb.2020.591358

**Published:** 2020-12-03

**Authors:** Marika Truu, Hiie Nõlvak, Ivika Ostonen, Kristjan Oopkaup, Martin Maddison, Teele Ligi, Mikk Espenberg, Veiko Uri, Ülo Mander, Jaak Truu

**Affiliations:** ^1^Institute of Molecular and Cell Biology, University of Tartu, Tartu, Estonia; ^2^Institute of Ecology and Earth Sciences, University of Tartu, Tartu, Estonia; ^3^Institute of Forestry and Rural Engineering, Estonian University of Life Sciences, Tartu, Estonia

**Keywords:** drained peatland forests, soil prokaryotic community, N-cycling genes, nitrogen gas emission, plant root biomass

## Abstract

Peatlands are unique wetland ecosystems that cover approximately 3% of the world’s land area and are mostly located in boreal and temperate regions. Around 15 Mha of these peatlands have been drained for forestry during the last century. This study investigated soil archaeal and bacterial community structure and abundance, as well as the abundance of marker genes of nitrogen transformation processes (nitrogen fixation, nitrification, denitrification, and dissimilatory nitrate reduction to ammonia) across distance gradients from drainage ditches in nine full-drained, middle-aged peatland forests dominated by Scots pine, Norway spruce, or Downy birch. The dominating tree species had a strong effect on the chemical properties (pH, N and C/N status) of initially similar Histosols and affected the bacterial and archaeal community structure and abundance of microbial groups involved in the soil nitrogen cycle. The pine forests were distinguished by having the lowest fine root biomass of trees, pH, and N content and the highest potential for N fixation. The distance from drainage ditches affected the spatial distribution of bacterial and archaeal communities (especially N-fixers, nitrifiers, and denitrifiers possessing *nosZ* clade II), but this effect was often dependent on the conditions created by the dominance of certain tree species. The composition of the nitrifying microbial community was dependent on the soil pH, and comammox bacteria contributed significantly to nitrate formation in the birch and spruce soils where the pH was higher than 4.6. The highest N_2_O emission was recorded from soils with higher bacterial and archaeal phylogenetic diversity such as birch forest soils. This study demonstrates that the long-term growth of forests dominated by birch, pine, and spruce on initially similar organic soil has resulted in tree-species-specific changes in the soil properties and the development of forest-type-specific soil prokaryotic communities with characteristic functional properties and relationships within microbial communities.

## Introduction

Peatlands are unique wetland ecosystems that cover approximately 3% of the world’s land area and are mostly located in boreal and temperate regions. Around 15 Mha of these peatlands have been drained for forestry in the past century (Paavilainen and Päivänen, 1995): in Europe, approximately 30% of peatlands have been drained for this purpose (Vasander et al., 2003). Ditch networks built to lower the water table in the peat column substantially affect soil oxygen and water conditions in these ecosystems. Furthermore, because peatlands are important carbon stores, converting them to forestland affects carbon and nitrogen cycles at regional and global scales (Joosten and Clarke, 2002; Limpens et al., 2008; Gao et al., 2014; Uri et al., 2017).

Biogeochemical cycles in forest soils are mediated by microbial communities and the quantity and quality of soil organic matter significantly affect the abundance, structure, and activity of bacterial and archaeal communities in these heterogeneous habitats (Norman and Barrett, 2016). Compared to fungi, there is limited research on bacteria and archaea in the soils of temperate and boreal forests (Sun et al., 2014; Baldrian, 2016; Ostonen et al., 2017; Truu et al., 2017). These studies are scarce also in the drained peatland forests. Sun et al. (2014) demonstrated that pine and spruce forests growing on boreal peat soils had high bacterial diversity and species richness related to the forest type. Studies investigating relationships between the soil bacteria, ectomycorrhizal fungi, and tree roots in forest ecosystems have shown that these systems simultaneously adapt to the specific conditions along the geographical area of distribution of a dominant tree species (Ostonen et al., 2017) and respond to environmental changes at the site triggered by global climate change (Truu et al., 2017).

Nitrogen transformation has been intensively studied in agricultural soils, marine ecosystems, and wastewater treatment systems (Kuypers et al., 2018), but insufficient attention has been paid to forest ecosystems. Studies concerning forests have focused either on the effect of climate change on bacterial community biomass (Lupon et al., 2015), or analyzed bacterial community structure in relation to N-mineralization (Landesman and Dighton, 2010; Lupon et al., 2015), amino acid production and nitrification (Lupon et al., 2015), or denitrification (Truu et al., 2017). Complex studies analyzing the long-term impact of dominant tree species on the structure of both soil bacterial and archaeal communities and their potential to perform nitrogen transformation as well as assessing the relationship of greenhouse gas (GHG) emission with microbial community parameters in the forest ecosystems are lacking.

The understanding of nitrogen fixation, nitrification, and denitrification is constantly expanding (Jones et al., 2013; Daims et al., 2015; van Groenigen et al., 2015; van Kessel et al., 2015; Koch et al., 2019), and completely new transformation pathways (Stein and Klotz, 2016) and organisms encoding several nitrogen-cycling (N-cycling) pathways (Nelson et al., 2016) have been discovered.

Nitrification, which is the sequential aerobic oxidation of ammonia via nitrite to nitrate, was considered to be a process where each step was mediated by specific microbial genera. However, recent findings confirmed the presence of complete nitrifying organisms (comammox) among species of the nitrite-oxidizing bacterial (NOB) genus *Nitrospira* (reviewed by Koch et al., 2019). The role of this process in forest ecosystems is still poorly studied (Wang et al., 2019).

Denitrification is a major N-reduction pathway that causes nitrogen losses from forest soils (Fang et al., 2015; Morse et al., 2015). Depending on the environmental conditions, nitrogen is reduced through several steps to the nitrous oxide (N_2_O) or dinitrogen gas (N_2_) during this process (Zumft, 1997). Denitrification can be used for respiration by dissimilatory nitrate reducers in anoxic environments or for nitrogen assimilation by microorganisms from all three domains of life (Maeda et al., 2015; Kuypers et al., 2018). Each step of this process is catalyzed by a specific reductase encoded by a specific gene (Zumft, 1997; Kuypers et al., 2018). The end-product is dependent on the presence of the gene encoding nitrous oxide reductase (*nosZ*) in the genome of a particular denitrifying organism. Only one-third of denitrifiers possess a complete denitrification pathway; the other two-thirds lack the *nosZ* gene and, consequently, the end product of the process is GHG N_2_O (Jones et al., 2008). The expression of these genes is highly dependent on the soil physiochemical properties including concentrations of substrate and inhibitors (Kuypers et al., 2018).

The potential of N-cycle processes is often evaluated by the abundance of marker genes in the environment. For example, nitrite reductase-encoding *nir* genes are used as markers to detect the genetic potential of denitrification because this step distinguishes denitrifiers from other nitrate-respiring bacteria (Zumft, 1997). The two types of *nir* genes (*nirS* and *nirK*) differ in their primary structure; are usually, with a few known exceptions, not harbored by the same organism (Graf et al., 2014); and are often utilized in different environmental niches (Hallin et al., 2009; Philippot et al., 2009; Ligi et al., 2014). Two different clades of *nosZ* genes are known, which encode reductases utilizing different enzyme transport pathways (Lee et al., 2006; Sanford et al., 2012; Jones et al., 2013). Organisms possessing *nosZ* clade I genes tend to colonize different habitats to those preferred by the denitrifiers that possess clade II genes (Graf et al., 2014; Ligi et al., 2015). Approximately 10% of the clade I and 30% of the clade II possessing organisms lack the genes from other denitrification steps making them potential sink of N_2_O (Graf et al., 2014).

The *amoA* gene, encoding the ammonium monooxygenase alpha subunit, is a widely used marker for evaluating the potential of the oxidation of ammonium or ammonia to nitrate. Although both bacteria and archaea can possess *amoA*, the gene is only distantly related between these organisms (Stahl and de la Torre, 2012; Alves et al., 2018). Both nitrification and denitrification can lead to nitrogen losses from soil, but there are at least two processes known to conserve this nutrient in the ecosystem: dissimilatory nitrate reduction to ammonia (DNRA) and nitrogen fixation (Tiedje, 1988; Sanford et al., 2012; Mania et al., 2014; van Groenigen et al., 2015). Widely used marker genes for these processes are *nrfA*, which encodes a nitrite reductase that catalyzes a key step, the formate-dependent nitrite reduction to ammonia in DNRA, and *nifH*, which encodes a nitrogenase subunit in nitrogen fixation (Reed et al., 2011; Welsh et al., 2014). Although the relevance of archaea to N-cycling, especially in the nitrification process in certain soils, has been highlighted (Prosser and Nicol, 2008; Alves et al., 2018; Espenberg et al., 2018), there remains a large gap in our knowledge about predominant gene clades and their functional role in nature (Alves et al., 2018).

This study investigated the effect of dominant tree species and drainage on soil bacterial and archaeal community composition and their functional properties in relation to N-cycling, in drained Histosol of middle-aged Downy birch, Scots pine, and Norway spruce forests. Furthermore, the relationship of forest soil microbial community structure and abundance with fine root biomass and turnover of trees as well as nitrogen gas fluxes were assessed in this study.

## Materials and Methods

### Description of the Study Sites

Nine middle-aged forests with a long drainage history and growing on Histosol in the Järvselja experimental forest district of southeastern Estonia were selected as study sites. Three stands dominated by Downy birch (*Betula pubescens*), three stands by Norway spruce (*Picea abies*), and three stands by Scots pine (*Pinus sylvestris*) were selected. Birch and spruce stands were classified as *Oxalis* fully drained site type, and pine stands as *Vaccinum* fully drained site type (Lõhmus, 1984). All studied stands were drained in the 1970s using open ditches and were unmanaged before the establishment of the sampling plots. Although the number of trees in the pine stands was significantly lower than in the birch and spruce stands, the basal area of the stands did not differ between the birch, pine, and spruce forests. Stand properties are described in Table 1.

**TABLE 1:** Geographical coordinates and characteristics of the stands, including the means and SD of soil characteristics of the four sampling plots of each studied stand (S, Norway spruce; B, Downy birch; P, Scots pine).

Stand	Geographical coordinates	Stand characteristics					
		Age (yr)	Area (ha)	Peat depth (cm)	Soil bulk density (g/cm)	Soil temp. (°C)	Soil water content (m3/m3)
**B1**	N 58°18′24,8 E 27°15′23,1	35	1.8	42 ± 3	0.14	11.8 ± 3.5	0.21 ± 0.13
**B2**	N 58°17′21,4 E 27°19′3,2	40	2.7	87 ± 2	0.17	12.0 ± 4.9	0.51 ± 0.18
**B3**	N 58°18′37,0 E 27°21′11,8	30	5.1	76 ± 5	0.15	11.8 ± 4.2	0.25 ± 0.11
**P1**	N 58°18′24,2 E 27°19′54,1	60	1.3	38 ± 13	0.10	11.6 ± 4.1	0.21 ± 0.10
**P2**	N 58°18′11,7 E 27°11′44,6	70	2.3	40 ± 8	0.14	11.0 ± 3.1	0.23 ± 0.11
**P3**	N 58°18′3,1 E 27°11′44,0	75	1.9	53 ± 11	0.12	11.2 ± 3.4	0.3 ± 0.18
**S1**	N 58°18′6,3 E 27°16′54,0	55	1.0	49 ± 3	0.11	11.7 ± 3.6	0.32 ± 0.14
**S2**	N 58°17′49,3 E 27°14′53,4	58	0.9	35 ± 10	0.25	10.8 ± 3.6	0.24 ± 0.15
**S3**	N 58°15′14,5 E 27°17′44,2	50	1.2	69 ± 14	0.10	11.2 ± 3.8	0.19 ± 0.10

*In stand P3, five plots were studied.*

In each stand, a transect with four sampling plots at distances of 5, 15, 40, and 80 m from the drainage ditch was established in April 2013. The stands are coded in this manuscript as B1–B3 for birch forests, P1–P3 for pine forests, and S1–S3 for spruce forests. The subscript number in the code indicates the plot distance (in meters) from the drainage ditch. At each sampling plot, the dynamics of groundwater table depth in the peat layer was measured using perforated polyvinyl chloride pipes (Ø 75 mm, sealed in the lower end) as monitoring wells. Soil temperature was measured with a temperature logger (Comet Systems Ltd., Rožnov pod Radhoštem, Czech Republic); electric conductivity and soil water content (SWC) were recorded in the 0–10 cm layer using a ProCheck reader equipped with a GS3 sensor (Decagon Devices Inc.).

### Collection and Analyses of Soil and Plant Root Samples and Measurement of Gas Emissions

Thirty-six composite soil samples were collected from 0–10 cm topsoil at all study sites in November 2014. Twelve subsamples were taken from around the gas chambers of each sampling plot to obtain the composite soil samples. Samples were kept in cool conditions during transportation to the laboratory, where they were then thoroughly mixed and divided into subsamples for chemical analyses and DNA extraction. Samples for chemical analyses were stored at 4°C and microbiological samples were stored at −20°C. Additionally, an intact soil core from the 0–10 cm soil layer of each sampling site was collected into a stainless steel cylinder (Ø 6.8 cm) during the sampling session and used for analysis of gas emission (N_2_, and N_2_O) in the laboratory. Cylinders were airtightly sealed and kept at cool conditions (4°C) until analysis. Detailed descriptions of plant root and gas sampling and analyses are provided in the Supplementary Material (S).

### DNA Extraction, PCR Amplification, and Sequencing

Total community DNA was isolated using a PowerSoil DNA Isolation Kit (Mo Bio Laboratories Inc., Carlsbad, CA, United States) according to the manufacturer’s instructions. The homogenization step was performed at 5000 rpm for 20 s using Precellys^®^ 24 (Bertin Technologies, France). Extracted DNA was stored at −20°C until downstream analyses. The quantity and quality of extracted DNA were determined by spectrophotometry (Infinite M200, Tecan AG, Austria).

Soil microbial community taxonomic profiling was performed using Illumina^®^ MiSeq sequencing of combinatorial sequence-tagged PCR products. Universal primers 515F (5′-GTGYCAGCMGCCGCGGTAA-3′) and 926R (5′-CCGYCAATTYMTTTRAGTTT-3′) (Parada et al., 2016) were used to target the V3-V5 hypervariable region of both the bacterial and archaeal 16S rRNA genes. The PCR mixture for amplification of each sample contained a unique combination of primers; each primer had specific 6-bp barcode sequence at the 5′ end (Parameswaran et al., 2007). Sequence data were deposited at the European Nucleotide Archive under project number PRJEB38904. Detailed descriptions of PCR conditions, sequencing, and bioinformatic analyses are provided in the Supplementary Material.

### Quantitative PCR Conditions and Data Analysis

Quantitative PCR (qPCR) was used to measure gene copy numbers in the studied soils. Bacterial and archaeal community abundances were evaluated using specific 16S rRNA genes (B16S and A16S, respectively). Nitrification potential was estimated using bacterial-, archaeal-, and comammox-specific *amoA* genes (*BamoA*, *AamoA*, and *CamoA*, respectively). Denitrification potential in the soil was evaluated by examining abundances of *nirS* and *nirK* genes, while N_2_O reduction potential was measured using *nosZ* clade I and II abundances (*nosZI* and *nosZII*, respectively). DNRA potential and nitrogen fixation potential were evaluated using *nrfA* and *nifH* abundance, respectively. QPCR assay details are supplied in the Supplementary Material (incl. Supplementary Table 1).

### Data Analyses

Principal component analysis (PCA) and between-group PCA were performed on soil physicochemical data using the R package ade4 v. 1.7-4 (Thioulouse and Dray, 2007). To assess the significance of differences between forest groups with respect to physicochemical variables, multivariate linear models were constructed. A model-based approach for the analysis of multivariate abundance data (MVANOVA) of microbial groups and gene abundance values with respect to forest type and distance from the ditch was applied using the anova function in R package mvabund v. 3.11.9 (Wang et al., 2012). Ordination of soil microbial communities was produced using edge PCA (edgePCA), taking advantage of the underlying phylogenetic structure of the data (Matsen and Evans, 2013). Two α-diversity indices were calculated. Inverse Simpson’s diversity index (1/S) was obtained based on the operational taxonomic unit (OTU) relative abundance table. Abundance-weighted phylogenetic diversity measure (BWPD) with θ = 0.5 was obtained according to McCoy and Matsen (2013). In the case of edgePCA and BWPD0.5 calculations, bacterial and archaeal reads from the soil samples were placed on a 16S rRNA gene tree created from The All-Species Living Tree project data (Munoz et al., 2011). Differences in microbial community structure between forest types were assessed using permutational multivariate analysis of variance (PERMANOVA) with 9999 permutations, implemented in the R package Vegan v. 2.4-1 (Oksanen et al., 2016). To identify soil physicochemical variables explaining variation in microbial community structure, distance-based regression analysis (db-RDA) with the Bray-Curtis dissimilarity matrix was applied using the DISTLM program with the forward selection procedure and 9999 permutations (McArdle and Anderson, 2001). Network analysis was performed using the SPIEC-EASI (SParse InversE Covariance Estimation for Ecological Association Inference) method (Kurtz et al., 2015).

Redundancy analysis (RDA) implemented in CANOCO 4.52 (ter Braak and Smilauer, 2002) was applied to assess the relationship between the abundance of N-cycling related genes and microbial genera estimated abundances. The forward selection method with the Monte Carlo test (999 permutations) was used to select the microbial genera relevant for the explanation of the variation in the abundances of N-cycling related genes. To overcome the problem with dimensionality in RDA, a set of predictor microbial genera for each gene was produced by random forest regression (RFR) prior RDA. Obtained lists of microbial genera were combined into a single dataset that was used as an input in RDA. The RFR was also used to select microbial genera that explain the variation in nitrogen gas emission values.

Heatmaps were produced using the ClustVis program (Metsalu and Vilo, 2015). For heatmaps showing clustering of stands according to the estimated nitrifying genera abundances, the estimates were obtained from bacterial and archaeal 16S RNA gene abundance values and proportions of genera in the whole bacterial or archaeal sequence sets, respectively.

## Results

### Root Parameters, Soil Chemical Characteristics, and Gas Emissions From the Studied Soils

**FIGURE 1:**
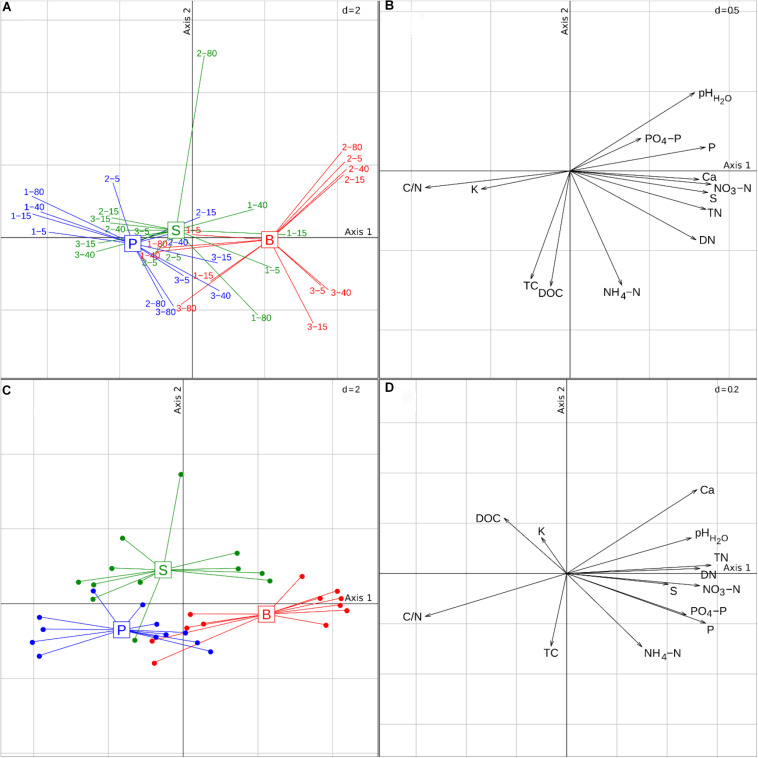
Ordination plots of the soil samples based on principal component analysis (PCA) and between-group PCA of the soil chemical variables. Scatterplots of samples **(A)** according to two first principal axes are shown, and correlation of soil chemical parameters with these axes **(B)**. Plots **(C,D)** are scatterplots of samples according to two first principal axes and correlation of soil chemical parameters with these axes in the case of between-group PCA. Samples are connected with lines to the respective group centroid. The first two principal components explain 49.4 and 16.2% of overall data variation, respectively. Abbreviations: B, birch forest; P, pine forest; S, spruce forest; abbreviations of soil chemical variables are given in Table 2.

At the time of sampling, the peat layer was on average 25 cm thinner in the pine forests than in the birch and spruce stands (Table 1). During the 2014 growing season (before soil sampling), the water level was below the peat layer in all studied stands and the soil moisture content in all three forest types was similar. There was no apparent moisture gradient in the soils of different forest types along the distance from the ditch. Although there was large variation in soil chemical composition between stands of the same forest type, soils of birch stands differed significantly from soils of pine and spruce stands [*p* < 0.01 and *p* < 0.05, respectively (Figures 1A,B and Supplementary Table 2)]. The three forest types differed in their soil pH, C/N ratio, and the contents of total nitrogen (TN), DN, NO_3_-N, total phosphorous (TP), PO_4_-P (*p* < 0.001 in all cases), and TS (*p* < 0.05). According to between-group PCA results, dominant tree species explained 23.6% of the variation in soil chemical variables (Figures 1C,D); however, no significant correlations between the soil chemical composition and distances from the drainage ditch were found in any of the studied stand types.

Fine root biomass of trees (FRBt) in the pine forests was on average twofold lower in the 0–10 cm soil layer compared with that of the birch and spruce forests (ANOVA, *p* < 0.001 in both cases; Supplementary Table 3). The ratio of FRBt to total (trees + understory) fine root biomass (FRBt/totFRB) was significantly lower in the pine stands than in the spruce stands (*p* < 0.01) but did not differ between the spruce and birch forests. There was a significant increase in the proportion of tree fine roots along the distance gradient in the pine and birch forests (*p* < 0.05 in both cases). Fine root TR was 1.6- and 1.3-fold higher in birch forests than in pine and spruce forests, respectively, and the difference in TR between birch and pine forests was significant (*p* < 0.001). TR was not significantly related to the distance from the drainage ditch in all studied forests.

*In situ* gas measurements indicated the highest but most variable N_2_O emissions were from the birch stand forest soils (*p* < 0.01 and *p* < 0.05 compared with pine and spruce stands, respectively) (Supplementary Table 3). Laboratory measurements indicated that N_2_O consumption occurred in the 0–10 cm soil layer of all birch sites as well as in most sites of the coniferous forests. Statistical analyses confirmed the difference in N_2_O consumption between deciduous forest and coniferous forests (*p* < 0.001 in both cases), while the coniferous forests did not differ significantly between each other in this respect. Similar to the N_2_O emissions, the amount of N_2_ emitted from the upper layer soils of birch forests significantly exceeded N_2_ emissions from soils of coniferous forests (*p* < 0.05 in both cases). Distance of the sampling site from the drainage ditch did not have a significant effect on N_2_O and N_2_ emissions in all studied forests.

### Abundance and Community Structure of Soil Prokaryotes

#### Bacterial 16S rRNA Gene Abundance and Community Structure

**FIGURE 2:**
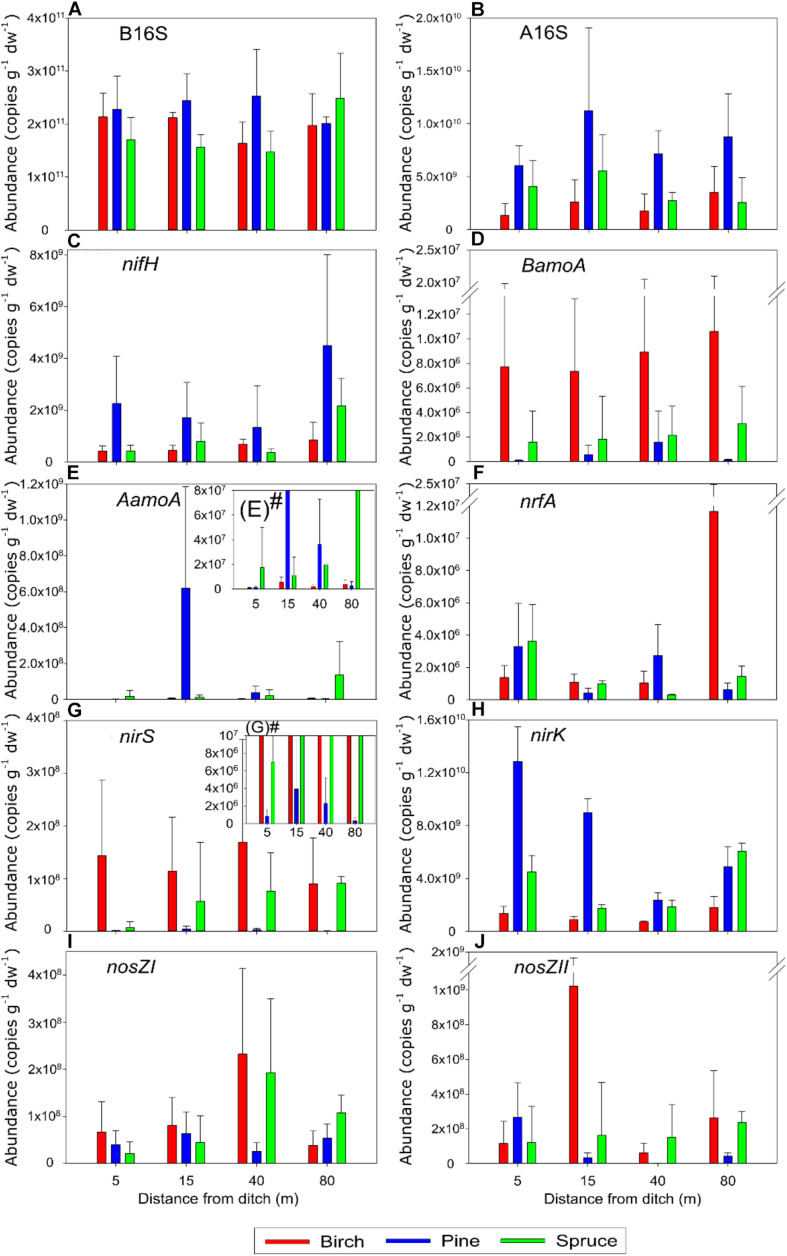
Mean ± SD of the abundances of the bacterial and archaeal 16S rRNA genes (B16S (plot **A**) and A16S (plot **B**), respectively) as well as the measured functional genes (plots **C–J**) of the three forest types. *BamoA*, bacterial *amoA*; *AamoA*, archaeal *amoA*. For the *AamoA* and *nirS* genes shown on plots **(E)** and **(G)**, respectively, that abundances varied highly between different forests, the lower range of abundances is presented on subplots **(E)**# and **(G)**#, respectively.

The B16S abundance was in the range of 10^11^ copies/g dry weight (dw) across all the study sites (Figure 2A and Supplementary Table 4), with no significant differences between forest types. Statistical analysis did not find any trends in B16S abundance values along the distance gradients of the studied forests. However, a small increase in mean abundance values from the ditch to the 40 m sites was notable in soils of pine forests; these values tended to decrease slightly along the same distance in the birch and spruce stands.

A total of 1,924,536 good quality 16S rRNA gene sequences were obtained for the prokaryotic community analysis after the quality check and denoising procedure. Of these sequences, 99.0% were phylogenetically affiliated with the domain Bacteria and clustered into 79,821 bacterial OTUs. The numbers of obtained sequences and OTUs, as well as the sequence coverage (SC) and diversity indices per plots of different forest types, are presented in Supplementary Table 5.

**FIGURE 3:**
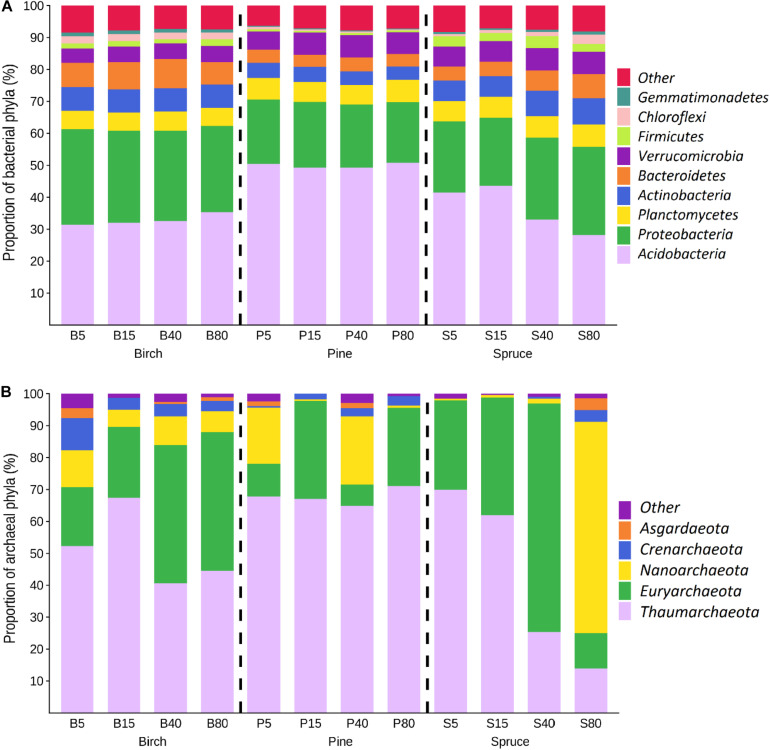
Mean proportions of the most abundant bacterial and archaeal phyla (**A,B**, respectively; *n* = 3) in soil samples of the studied stands. Numbers indicate plot distance (in meters) from drainage ditches. Groups with proportions <1% are summed and indicated as Others.

Across all study sites, the most abundant bacterial phyla were *Acidobacteria*, *Proteobacteria*, *Planctomycetes*, and *Bacteroidetes*, accounting for 76% of the bacterial community on average (Figure 3A). The stand type significantly affected the soil bacterial community structure at the phylum level (MVANOVA, *p* < 0.001). Among the dominant phyla, *Bacteroidete*s (4.0–8.2%), *Chloroflexi* (0.5–2.3%), and *Gemmatimonadetes* (0.3–1.2%) were more abundant in the birch stand soil communities than in the soil communities of coniferous forests (*p* < 0.001 in all cases). Statistically significant differences between the birch, pine, and spruce stands were also found for the proportions of some less abundant (<0.5%) phyla (*p* < 0.05 in all cases) (Supplementary Table 6) that decreased in the birch forests (*Rokubacteria* 0.38–0.22%) or increased in the spruce forest (*Rokubacteria* 0.09–0.40%, *Nitrospirae* 0.07–0.24%, and *Spirochaetes* 0.05–0.11%) or pine forests (FCPU426 0.18–0.31%) along the distance gradient.

**FIGURE 4:**
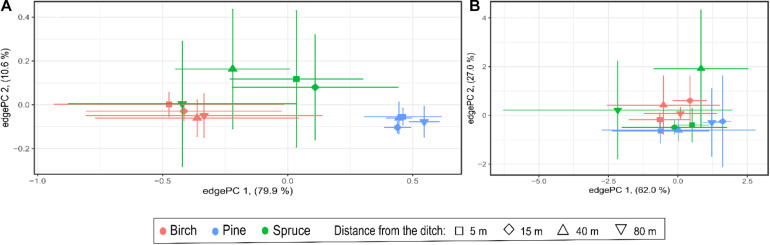
EdgePCA plots showing ordination of study plots according to bacterial and archaeal community phylogenetic similarity (**A,B**, respectively). Numbers indicate plot distance (in meters) from drainage ditches.

On the edgePCA plot, bacterial communities of pine forests formed a compact cluster that separated clearly from the much more dispersed cluster of birch forest communities (Figure 4A). Although the variation among bacterial communities of the birch stands was large, a general trend of change in community from the drainage ditch to the 40 m site was evident. Soil bacterial communities varied most in the spruce forest and these communities were separated from the other studied forests along the edgePCA axis 2. The distinction of the 80 m plot communities from those of the plots locating closer to the ditch was characteristic for the three types of forests studied.

The diversity index B1/S ranged from 45 to 310 across all the studied sites (Supplementary Table 5). Values were lower and less variable in the pine stands (72 ± 17) than in the birch and spruce forests (171 ± 83 and 152 ± 78, respectively). The difference in B1/S values was significant between the birch and pine stands (*p* < 0.001) and between the pine and spruce stands (*p* < 0.05). BWPD_*B*_ values were also highest (13.2 ± 0.6) in soil samples of the birch stands followed by soils of the spruce stands and then the pine stands (12.3 ± 0.6 and 11.2 ± 0.3, respectively). Differences in BWPD_*B*_ values were significant (ANOVA, *p* < 0.001) between all three forest types. No significant relationship between the plot distance and diversity index values was found in any of the studied forest types.

**FIGURE 5:**
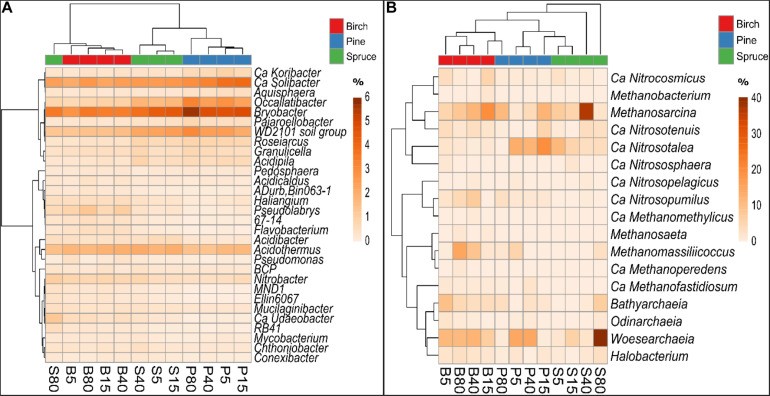
Heatmap showing clustering of the plots of birch (B), pine (P), and spruce (S) forests according to the average (*n* = 3) bacterial **(A)** and archaeal **(B)** genera proportions (%) in the soil bacterial and archaeal communities, respectively. The number in the plot code indicates plot distance (in meters) from a drainage ditch.

The heatmap based on the bacterial genera proportions showed a clear clustering of the soil samples according to the forest type (Figure 5A). There were 24 bacterial genera, including *Ca. Koribacter, Ca. Solibacter, Roseiarcus, Pedosphaera, Gemmata*, and *Nitrobacter*, that were significantly different (MVANOVA, *p* < 0.01 or *p* < 0.05) between the studied forest types (Supplementary Figure 1). Clustering of sampling plots according to proportions of genera in the soil bacterial community indicated that trends in bacterial genera variation along the distance gradient were forest-type specific. In the spruce forests, there was higher similarity in bacterial genera composition between the plots locating closer to the ditch and substantial distinction of these plots from the 80 m plots. In the pine forests, the 15 m plots could be distinguished from the two surrounding plots by bacterial genera composition, while in the birch forests, the two plots located closest to the ditch were distinguished from the two more distant plots.

In each of the studied forest types, a unique prokaryotic community network was formed (Supplementary Figure 2). These networks consisted of seven larger modules (≥5 OTUs) and included 31, 32, and 46% of the OTUs detected in the birch, pine, and spruce forest soils, respectively. The highest number of OTUs was involved in the network of the pine forests, but the modularity was the lowest compared to the two other obtained networks (Supplementary Table 7). Most of the other network properties (e.g., average connectivity, eigenvector, clustering coefficient, etc.) were more similar between the two coniferous forest types. The birch forest network consisted exclusively of the bacterial OTUs, while both of the coniferous forest networks also involved one archaeal OTU.

#### Archaeal 16S rRNA Gene Abundance and Community Structure

The A16S abundance (5.4 × 10^8^ to 2.0 × 10^10^ copies/g dw across all the studied forests) (Figure 2B and Supplementary Table 4) was significantly higher in the pine forests (8.3 × 10^9^ ± 4.5 × 10^9^ copies/g dw) than in the birch and spruce stands (2.3 × 10^9^ ± 4.5 × 10^9^ copies/g dw, *p* < 0.001 and 4.0 × 109 ± 2.6 × 10^9^ copies/g dw, *p* < 0.05, respectively), which did not differ significantly from each other. There were no clear trends in A16S abundance along the distance gradient in any of the forest types, but the abundances tended to be lowest in the plots 5 m from the drainage ditch in pine and birch forests. The proportion of A16S in the total prokaryotic 16S rRNA genes in the studied forests (0.4 to 6.18%) (Supplementary Table 8) was less variable and significantly lower in birch stands (1.1 ± 0.8%) compared with the pine stands where the A16S proportion was the highest (3.5 ± 1.6%, *p* < 0.001). The difference in proportion of A16S between the pine and spruce stands (2.4 ± 1.8%) was also significant (*p* < 0.05). Along the distance gradient, the A16S proportion was lowest in the 5 m plots of birch stands; however, this was not the case in the coniferous forests. A clear trend of an increase in A16S proportion along the distance gradient was only observed for the P2 stand.

Of the good quality sequences, 0.36% were phylogenetically affiliated with the domain Archaea and clustered into 225 OTUs. Archaeal sequence numbers, SC values, and diversity indices per plots of different forest groups are presented in Supplementary Table 5.

Similar to the bacterial community, the highest within-site variation in the archaeal community composition was observed in the spruce forests. Five archaeal phyla were identified (Figure 3B); the proportion of unclassified archaeal sequences was between 0% (P_15_) and 4.6% (B_5_). *Thaumarchaeota* was the dominant archaeal phylum in almost all studied soils with proportions of 24–60, 40–91, and 6–82% in the soil archaeal communities of birch, pine, and spruce stands, respectively. The exceptions were sites where the phyla *Nanoarchaeota* (plots P1_40_ and S2_80_) and *Euryarchaeota* (plots B2_40_, B1_80_, S3_5_, S3_15_, and S2_40_) dominated. A decreasing trend in the proportion of *Thaumarchaeota* (on average from 70 to 14%) across the distance gradient was detected in the spruce forests. The proportion of the phylum *Crenarchaeota* was highest in the birch forest archaeal communities (5.2 ± 3.2%), and there was a general trend of a decrease in this proportion (10–2.3%) along the distance gradient. In the coniferous forests, *Crenarchaeota* was detected in three spruce forest plots (3% in S3_40_, 2.6% in S1_80_, and 11.5% in S2_80_) and in seven pine forest plots (1.0–7.9%) with no distinct distribution pattern.

On the edgePCA plot, the clustering of plots based on archaeal community structure was not as distinctive as that observed for the bacterial community; additionally, there were no visible trends along the distance gradient (Figure 4B). Clustering of the samples according to forest type and higher similarity between the plots locating closer to the ditch was evident in the heatmap based on archaeal genera proportions (Figure 5B).

Significantly higher A1/S values were obtained for the birch forest soils (11.7 ± 6.5) than for the pine forest soils (6.2 ± 2.2; *p* < 0.001) while differences between the spruce stands (7.6 ± 4.8) and the pine and birch forests were not significant (Supplementary Table 5). BWPD_*A*_ was highest in the birch stands (8.1 ± 1.6) followed by the spruce and pine stands (7.0 ± 2.4 and 5.3 ± 3.2, respectively). A significant difference in the BWPD_*A*_ values was only observed between the birch and pine forests (*p* < 0.001), while an increase in the mean value (from 6.2 to 8.8) of this index along the distance gradient was recorded for the spruce forests.

Archaeal OTU involved in the networks of pine and spruce forests (Pm4 and Sm7, respectively) belonged to the class *Thermoplasmata* in the phylum *Euryarcheota* (Supplementary Figures 2B,C).

#### Relationship Between Site-Specific Factors, Microbial Community Structure, and 16S rRNA Gene Abundances

Across all the stands, the peat depth (PD) and its NH4 concentration were significantly related to B16S abundance in the upper 10 cm soil layer, while several chemical factors, including pH, TC content, and content of TN and its dissolved fractions, as well as the tree root traits (FRBt and TR), correlated with A16S abundance in the studied soils (Table 2). The SWC affected the 16S rRNA gene abundances only in spruce forests (*r* = 0.75, *p* < 0.01).

**TABLE 2:** Statistically significant Pearson correlations between the gene abundances and soil characteristics and fine root traits in the studied forest soils (*n* = 37).

Variable	Gene abundances									
	B16S	A16S	*BamoA*	*AamoA*	*CamoA*	*nirS*	*nirK*	*nosZI*	*nosZII*	*nifH*
pH		−0.71***	0.71***	0.33*	0.84***	0.87***		0.65***	0.63***	
TC		0.45**			−0.43**	−0.41*				
TN		−0.41*	0.56***	0.40*	0.51**	0.54**	−0.48**	0.44**	0.33*	
DN		−0.51**	0.75***	0.42**	0.72***	0.64***		0.58***	0.56***	
NH_4_	0.44**		0.34*	0.35*						0.40*
NO_3_		−0.52**	0.82***	0.52**	0.84***	0.79***	−0.35*	0.62***	0.58***	
TP		−0.45**	0.59***	0.34*	0.62***	0.61***	−0.43**	0.47**	0.44**	
PO_4_		−0.40*			0.42**	0.39*	−0.35*			
S		−0.35*	0.52**	0.50**	0.51**	0.55***		0.54**	0.36*	
K			−0.34*	−0.69***				−0.34*	−0.35*	
Ca		−0.65***	0.77***	0.43**	0.77***	0.84***	−0.41*	0.70***	0.57***	
C/N		0.54**	−0.58***	−0.43**	0.61***	−0.65***	0.40*	−0.54**	−0.40*	
FRBt		−0.47**	0.33*		0.40*	0.33*	−0.34*			−0.38*
FRB_*t*_/totFRB			0.33*							
TR		−0.40*								
SWC			0.35*	0.42**				0.40*	0.36*	0.41*
PD	−0.41*	−0.57**				0.37*				

B16S, bacterial 16S rRNA gene; A16S, archaeal 16S rRNA gene; BamoA, bacterial amoA; AamoA, archaeal amoA; CamoA, comammox-specific *amoA*; FRBt, trees fine root biomass; FRB_*t*_/totFRB ratio between trees fine root biomass and total (trees + understory) fine root biomass; TR, root turnover rate per year; TC, total carbon; TN, total nitrogen; DN, dissolved nitrogen; TP, total phosphorous; SWC, soil water content; PD, peat depth. *p < 0.05; **p < 0.01; ***p < 0.001.

The db-RDA indicated a weak effect of SWC on the community variation while the soil chemical composition had a strong forest type-dependent effect on the community structure of the soil prokaryotes. Almost 78% of prokaryotic community variation was explained by the pH and contents of NH_4_, NO_3_, PO_4_, TC, K, and Ca, in soil across all the studied forests (Supplementary Table 9); the pH alone explained most of the variation while the individual contribution of the other factors was ∼2–3%. The effect of chemical factors was dissimilar in different types of forests. The NH_4_ and K concentration in the soils of birch forests, pH and C/N in the pine forests, and pH, TN, TP and Ca in the spruce forests explained 58, 71, and 92% of the community variation, respectively. Furthermore, the effect of soil chemistry was not similar in the case of the modules of the microbial ecological networks of different forest types. In the birch forests, only the K content was a significant factor explaining 40–44% of the variation in the structure of three modules, while in the pine and spruce forests all the obtained modules were related to at least one, but in most cases more factors. All these sets of relationships were specific for each module and explained 38–78 and 83–93% of the variation in the pine and spruce modules, respectively. From the measured plant root traits, the FRBt had the highest impact on the abundance and community structure of prokaryotic organisms in the studied soils (Table 2 and Supplementary Table 9). This factor was negatively related to the abundance of A16S and explained almost 17% of the community variation across all the stands. In the pine and spruce forest soils, 25.7 and 35.0% of the whole community, as well as more than 35 and 25% of the structure of several network modules, respectively, was explained by the FRBt, but in the birch forest soils, there was no evidence of this effect.

### Abundance and Proportion of N-Cycling Organisms

#### N-Fixation

The *nifH* gene was detectable in soils of all study sites and the abundance ranged from 6.3 × 10^7^ to 7.9 × 10^9^ copies/g dw (0.05 to 3.77% of the B16S) across the sites (Figure 2C and Supplementary Tables 4,8). The abundance of *nifH* was significantly higher in the soils of pine stands than in the other two forest soil groups (ANOVA, *p* < 0.05), which did not differ from each other in this respect. The proportion of *nifH* in the bacterial community, although variable between the stands of each forest type, was significantly higher in the pine forest soils (1.04 ± 1.06%) than in the birch forest soils (0.30 ± 0.18%, *p* < 0.05) but did not differ between the birch and spruce stands. Across all the pine forest soils, *nifH* abundance and proportion in the bacterial community tended to decrease from the drainage ditch to the 40 m plots; the highest values were recorded in soils of plots that were 80 m from the drainage ditch. The increase in proportion of *nifH* along the distance from the ditch across all the studied forests was statistically significant (MVANOVA, *p* < 0.05).

#### Nitrification

*BamoA* was detected in all samples, with abundance values ranging from 2.3 × 10^4^ to 2.2 × 10^7^ copies/g dw (Figure 2D and Supplementary Table 4). *BamoA* comprised 0.0001–0.0041% of the B16S gene abundance in the coniferous forests as well as in the birch stand B1. In the other two birch stands, the proportion of this gene was as high as 0.01% (Supplementary Table 8). The mean value of *BamoA* abundance was considerably high in most sampling points of the birch stands and was lowest in the pine forests; however, because *BamoA* abundance also showed large variation in the stands of a forest type, no statistical differences were found between the forest types. Furthermore, there were no clear trends along the distance gradient in terms of either the abundance of this gene or in its proportion in soil.

Quantitative PCR confirmed the presence of a *CamoA* gene in all plots of B2 and B3 stands (8.7 × 10^4^–4.5 × 10^6^ copies/g dw). These genes formed 0.01–0.76% of the estimated *Nitrospira* 16S rRNA gene abundance in the soil, and in both stands, the proportion of this gene followed a similar spatial pattern, being significantly higher in the 5 and 40 m plots (0.38–0.76%) than in the 15 and 80 m plots (0.01–0.16%) (Supplementary Tables 4,8). *CamoA* was also detected in two spruce forests (1.7 × 10^6^–3.6 × 10^6^ copies/g dw). In most plots of the birch forests and in all S1 plots, the abundance of *CamoA* was significantly lower than that of *BamoA* in the same soil, and accounted for 1.8–18 and 1.3–5.5% of all the *amoA* genes in the birch and spruce stands, respectively. However, there were two plots (B2_5_ and S2_80_) where *CamoA* abundance exceeded *BamoA* abundance by approximately 3.4-fold and comprised 65.1 and 55.8% of all *amoA* genes in these soils, respectively.

*AamoA* was also detected in all study sites and the abundance of this gene was more variable (from 8.7 × 10^3^ to 1.1 × 10^9^ copies/g dw) between the sampling sites compared with the abundance of *BamoA* (Figure 2E and Supplementary Table 4). The proportion of *AamoA* was 0.01–7.6% of the A16S abundances in the studied soils (Supplementary Table 8). Like *BamoA*, the abundance of *AamoA* did not differ significantly between the forest groups and was not related to the plot location at the site. However, the S1 stand had a significantly higher proportion of *AamoA* (2.8 ± 2.4%) than the other two studied spruce stands (0.09 ± 0.10 and 0.008 ± 0.009% in S2 and S3, respectively), and the spruce forests all had the lowest *AamoA* abundances in the plots located 15 m from the drainage ditch.

**FIGURE 6:**
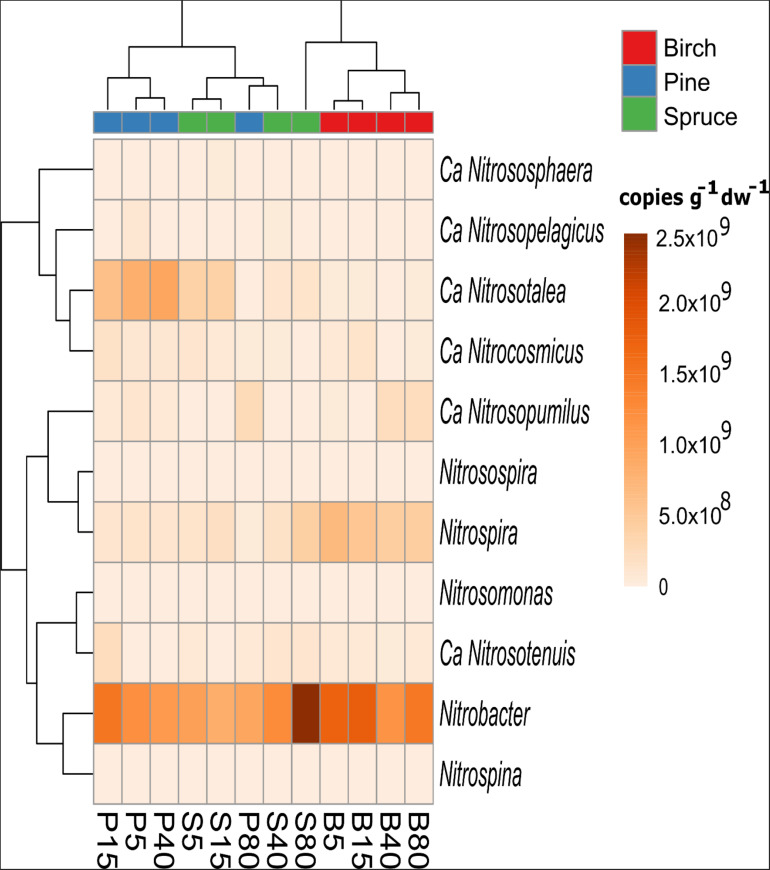
Heatmap showing clustering of the study plots according to the average estimated abundances of nitrifying bacterial and archaeal genera in the birch (B), pine (P), and spruce (S) forest soils (*n* = 3). The number in the plot code indicates plot distance (in meters) from a drainage ditch.

Based on the taxonomic affiliation of 16S rRNA gene sequences, organisms from two genera of autotrophic ammonia-oxidizing bacteria (AOB; *Nitrosomonas* and *Nitrosospira*) and three genera of NOB (*Nitrobacter, Nitrospira*, and *Nitrospina*) were detected. In the case of AOB, *Nitrosospira* dominated in all forests and the proportion of this genus in the soil bacterial community did not differ significantly between forest types. However, the proportion of *Nitrosospira* in the community did decrease with increasing distance from the drainage ditch in birch and spruce forests (from 0.012–0.004 and 0.013–0.000%, respectively). With one exception (plot B2_5_), *Nitrobacter* was the dominant genus of NOB (0.80 ± 0.34, 0.69 ± 0.28, and 0.50 ± 0.10% in birch, spruce, and pine forests, respectively) (Figure 6). The proportion of the genus *Nitrospira* in the soil bacterial community was on average 1.3- and 1.2-fold higher in birch forest soils (0.31 ± 0.04%) compared with that in pine (*p* < 0.05) and spruce forests (*p* < 0.001), respectively (Supplementary Table 10). Nitrifying archaea from six genera, *namely Ca. Nitrosotalea, Ca. Nitrocosmicus, Ca. Nitrososphaera, Ca. Nitrosopelagicus, Ca. Nitrosopumilus*, and *Ca. Nitrosotenius* were found in the studied forest soils. Forest type and plot distance from the ditch, as well as their interaction, significantly affected the structure of the nitrifiers community in the studied soils (MVANOVA, *p* < 0.001 in all cases). *Ca. Nitrosotalea* was the predominant genus in the archaeal communities of birch and spruce forests, while in the pine forests, *Ca. Nitrocosmicus* (plots P_5_ and P_15_) or *Ca. Nitrosopumilus* (plots P_40_ and P_80_) dominated depending on the plot location. Plots of the same forest group locating closer to the drainage ditch (5 and 15 m plots in birch and spruce forests, and 5, 15, and 40 m plots in pine forests) grouped together also on the heatmap based on cluster analysis of the estimated abundances of both bacterial and archaeal nitrifiers genera, while communities of the two more distant plots in birch forests formed a separate cluster (Figure 6).

#### DNRA

The *nrfA* gene was detected in all soils of the studied sites. The abundance of *nrfA* ranged between 1.2 × 10^5^ and 2.6 × 10^7^ copies/g dw and formed 0.0001–0.0112% of the B16S gene abundance across all the studied forests (Figure 2F and Supplementary Tables 4,8). *NrfA* abundance differed between the stands of each forest group, but there were no significant differences in *nrfA* abundance between the forest types. However, there were some notable systematic trends in the birch and pine forests, including a decrease in *nrfA* abundance from the plots at 5 m to those at 40 m in the B1 and B3 stands, and a lower abundance of *nrfA* in the 15 m plots of the pine forest compared with the 5 and 40 m plots of this forest type.

Although the proportion of *nrfA* varied greatly between the stands of each forest type, there was a significant increase in the proportion along the distance gradient for the birch stands (*r* = 0.61, *p* < 0.05). In all the spruce forests, a decrease in *nrfA* proportion from the 5 m plots to the 40 m plots was observed. Thus, the plot distance from the ditch significantly affected *nrfA* proportion in the bacterial community across all the studied forests, and the effect was different in the birch and coniferous forests (MVANOVA, *p* < 0.001).

#### Denitrification

The abundance of the gene *nirS* ranged between 1.7 × 10^4^ and 3.8 × 10^8^ copies/g dw (Figure 2G and Supplementary Table 4) and formed 0.0001–0.20% of the B16S abundance in the soils of studied forests (Supplementary Table 8). The abundance of the *nirS* gene was unevenly distributed across the studied forests. For example, there was a large difference (2–3 orders of magnitude) in *nirS* abundance between stand B1 and the two other birch stands. No statistically significant differences in abundances of this gene were found between forest groups.

The proportion of *nirS* was highest in the bacterial communities of the B2 and B3 stands (0.13 ± 0.06 and 0.09 ± 0.01%, respectively) and lowest in the P1, P2, and S3 stands (0.0001 ± 0.0001, 0.0002 ± 0.0002, and 0.0002 ± 0.0003%, respectively). There were no clear trends in the proportion of *nirS* along the distance gradient from the drainage ditch in the studied forests.

The gene *nirK* was more abundant (from 6.1 × 10^8^ to 1.6 × 10^10^ copies/g dw) than the *nirS* gene in soils of the studied forests (Figure 2H and Supplementary Table 4) with proportions ranging from 0.28 to 5.99% in the soil bacterial communities of all the stands (Supplementary Table 8). *NirK* was more abundant in coniferous forests than in the birch forests (*p* < 0.001 in both cases). In all birch and spruce stands, *nirK* abundance was highest on the 80 m plots. In contrast, in the pine forest stands, maximum values were measured on the 5 m plots and minimum values in the 40 m plot soils. Statistical analysis confirmed a significant forest type-specific relationship between *nirK* abundance and the distance gradient in the studied forests (MVANOVA, *p* < 0.001). The *nirS*/*nirK* ratio varied between the stands of each forest type and was significantly lower in the pine forest soils (0.004 ± 0.001) than in soils of the spruce forests (0.021 ± 0.039, *p* < 0.05) and the birch forests (0.126 ± 0.140, *p* < 0.01); the difference between the latter two forest types was also significant (*p* < 0.05). In the birch forests, the ratio was always lowest in the 80 m plots, and in the pine forests, the ratio was always highest in the 40 m plots. However, such regularities were not observed in the spruce plots.

The abundance of *nosZI* was between 4.9 × 10^6^ and 3.6 × 10^8^ copies/g dw (0.002–0.19% of the 16S rRNA gene abundances) in soils across all the studied forests (Figure 2I and Supplementary Tables 4,8). *NosZI* abundance varied between the stands of a forest type, but neither the abundance nor the proportion differed between the studied forests. Although *nosZI* abundance and proportion were not related to the distance of the plot from the drainage ditch, similar trends were observed for the birch and spruce stands where both these parameters were always lowest in the 80 m plots. The proportion of *nosZI* increased from the 5 m plots up to the 40 m plots of these forests but was 2.3- to 9.4-fold lower in the 80 m plots than in the 40 m plots. In the pine forests, the abundance and proportion of *nosZI* were always lowest in the 40 m plots and higher in the 15 m plots compared to the 5 m plots.

*NosZII* abundance was more variable across all the stands, ranging from 1.9 × 10^6^ to 1.6 × 10^9^ copies/g dw, and the proportion was 0.002–0.73% in the bacterial communities of the studied forest soils (Figure 2J and Supplementary Tables 4,8). Both these parameters were not different between the forest types. Across all the forests, the highest abundance and proportions of *nosZII* were measured in the soils of plots B2_15_ and B3_15_, while the lowest values were obtained for the B1 stand. In all pine forests, *nosZII* abundance was highest in the 5 m plots and a decreasing trend in abundance up to the 40 m plots was visible; however, in the 80 m plots, the *nosZII* abundance was almost an order of magnitude higher than in the 40 m plots. No similar trends were detected for the three spruce stands in this respect.

The *nosZI*/*nosZII* ratio ranged from 0.07–14.67 across all the studied plots and did not differ significantly between the forest types. The ratio increased along with the increasing distance from the drainage ditch up to 40 m in all birch and pine forests where *nosZI* abundance always exceeded *nosZII* abundance.

The ratio between total *nir* and *nosZ* genes (*nir*/*nosZ*) ranged from 0.73 to 417 across all the studied sites. Like the other gene parameters, this ratio varied greatly between the stands of each forest group and was significantly different between different forests (MVANOVA, *p* < 0.05, Supplementary Tables 4,8). The ratio was highest and most variable (113 ± 113) in the soils of pine forests where the 15 and 40 m plots had always higher *nosZ/nir* ratios than the 5 and 80 m plots. In the birch forests, the ratio was much lower (30.0 ± 57.0) and the tendency within stands was vice versa. The ratio in spruce stands stayed between the two other groups (66.1 ± 74.2) and the 5 and 15 m plots of two spruce stands (S2 and S3) had from four- to 13-fold higher *nosZ/nir* than the rest of the plots of spruce forests.

### Relationship Between N-Cycling Gene Abundances, Soil Parameters and Microbial Community Structure

Soil chemical composition had a strong effect on the abundances of N-cycle functional genes in the studied soils (Table 2). The abundance of most genes (except *nirK* and *nifH*) was significantly related to the soil pH and contents of TN and its dissolved fractions, C/N, TP, S, and Ca. In the case of *amoA* genes, the pattern of relationships was similar for all the targeted *amoA* types. Firstly, pH and the concentrations of TN and TP, as well as their dissolved fractions, correlated with the abundances of these genes, but stronger correlations were almost always found for the two bacterial-specific genes (*BamoA* and *CamoA*) compared with the archaeal-specific gene (*AamoA*). Furthermore, *CamoA* was not detectable in soils with a pH_*H*__20_ value below 4.6 (Supplementary Figure 3).

For the *nir* genes, a higher number of significant relationships with chemical factors were revealed for *nirS* than for *nirK* and the correlations with parameters related to both genes were always opposites. Similar chemical factors were significant in the case of the two *nosZ* clades, but the correlations were always stronger for *nosZI*.

Across all the forests, SWC had an effect on the N-cycle functional genes (Table 2) but differences between forest types were again evident: *nrfA* abundance (*r* = −0.80, *p* < 0.01) in the birch forests and *BamoA*, *CamoA*, *nirS*, and *nosZII* abundances (*r* = 0.59–0.74, *p* < 0.01 in all cases) in the spruce forests were affected. A strong correlation between FRBt and abundances of N-cycling genes such as *nifH*, *BamoA*, *CamoA*, *nirS*, and *nirK* was found in the soils of drained forests.

**FIGURE 7:**
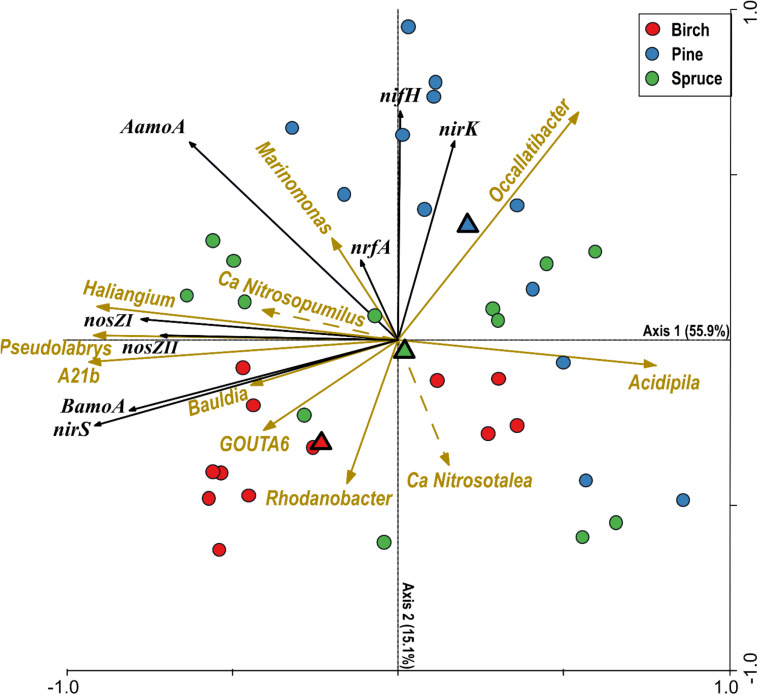
Ordination triplot based on the redundancy analysis (RDA) of the gene abundance data with respect to the microbial genera estimated abundances, displaying 71.0% of variance in the abundances and 88.7% of variance in the fitted abundances. All shown bacterial genera were significant (*p* < 0.05; 999 Monte Carlo permutation tests) and overall RDA solution was significant (*p* < 0.001, 999 Monte Carlo permutation tests). Two archaeal genera were added as passive variables to the RDA plot. Individual sample scores are shown as circles and stand type centroids are indicated by triangles. The length and direction of the solid and dashed lines indicate the approximate correlations between the ordination axis and different bacterial and archaeal genera, respectively.

Prior RDA, RFR was applied to produce a set of predictor microbial genera for each gene (Supplementary Figures 4–6) and these individual RFR results were combined into a single dataset. According to the results of RDA, the abundance of nine bacterial genera described 77.1% of the overall variation in the abundances of N-cycling-related genes (Figure 7). The highest values of explained variance were obtained for *nirS*, *BamoA* and *AamoA* (≥80%) and lowest for *nrfA* (43%) gene. Stand type alone explained 16.7% of the overall variation in the abundances of N-cycling-related genes while together the abundance of nine bacterial genera and stand type explained 80% of the overall variation. The RDA first axis was strongly correlated with the abundance of three bacterial genera (*Haliangium*, *Pseudolabrys*, A21b) and mostly explained variation in *nosZI* and *nosZII* abundances. The abundance of these three bacterial genera was not different between forest groups and correlated strongly with soil pH (*r* > 0.75, *p* < 0.01). RDA results indicate that *nirS* and *BamoA* abundances covaried strongly and were positively related to the abundances of genera *Bauldia* and GOUTA6. Abundance of these two genera was correlated in addition to pH to fine root biomass. The RDA second axis was mainly related to the variation in *nifH* and *nirK* abundances and this axis was positively correlated with the genus *Occallatibacter* abundance.

### Relationship Between the Microbial Community Structure and Nitrogen Gas Emissions

Across all the stands, a strong relationship was revealed between the soil microbial community diversity indices and both measured N_2_O emissions and sink; however, the correlation was negative in the case of *in situ* emission and positive for the N_2_O_10 *cm*_ and sink calculated for the 0–10 cm soil layer (Supplementary Table 11). In the case of N_2_ emission, only a weak negative correlation with BWPD_*B*_ was found.

In total, 62 prokaryotic genera were associated with the N_2_O_10 *cm*_ emission and 40 genera with the sink across all the sites. The top part of these two lists overlapped to a large extent as the same bacterial genera (vadinHA17, SB-5, and *Thiobacillus*) occupied the first places on both lists (Supplementary Figures 7B,C). Some archaeal groups also appeared to be related to the N_2_O_10 *cm*_ emission (*Methanosaeta, Bathyarchaeia*, and *Ca. Methanopereden*) and sink (*Methanomassiliicoccus*, Group 1.1c, *Ca. Nitrososphaera, Woesearchaeia, Ca. Methanofastidiosum, Methanosarcina*, and *Methanobacterium*) or to both these parameters (Marine Group II). The proportions of several genera related to these gas emissions were also significantly different in the communities of studied forest soils (Supplementary Table 12).

There were no correlations found between N_2_O_10 *cm*_ emission and gene parameters across all sites, but a relationship with the proportion of *nirK* in the birch forests and with the abundance and proportion of *nifH*, as well as the *nosZI* proportion and *nosZ/nir* ratio in the pine forests was found (Supplementary Table 11).

**FIGURE 8:**
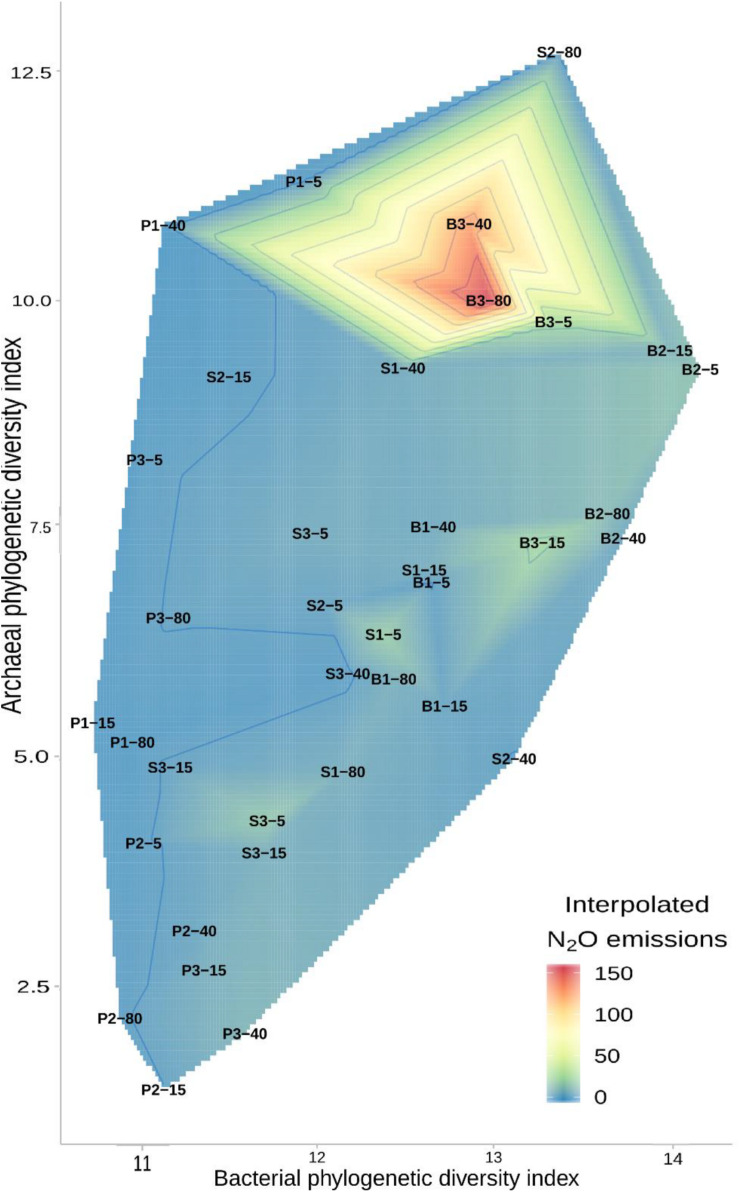
Contour plot showing the relationship between balance-weighted phylogenetic diversity indices for bacteria and archaea and the *in situ* N_2_O emission in the soils of birch (B), pine (P), spruce (S) forests. The first number of the plot code indicates the stand number and the second number indicates plot distance (in meters) from a drainage ditch.

Across all the studied forests, the *in situ* N_2_O emission was related to the bacterial and archaeal community diversity (Figure 8). The RFR analysis did not find any significant relationships between the estimated prokaryotic genera abundances and *in situ* N_2_O emission across all the sites, but correlations occurred in the pine forest soils, where 54 genera including the archaeal genera *Ca. Nitrosopumilus, Methanosarcina, Bathyarchaeia, Halobacterium*, and *Ca. Nitrosotenuis* were found to be related to this emission. The top 20 genera from this group explained 53% of the *in situ* N_2_O emission in these soils (Supplementary Figure 7B).

In spruce and birch soils where *CamoA* was detected, N_2_O emission was strongly related to *CamoA* abundance and proportions (*CamoA/B16S, CamoA/BamoA, CamoA/total amoA*). Several significant correlations between N_2_O emission and functional gene abundances and proportions (e.g., *BamoA*, *AamoA*, total *amoA*, *nirS*, *nirS*/*nirK*, and *nosZI*) were found in the coniferous forests; these relationships were often negative in the pine forests and positive in the spruce forests (Supplementary Table 11). *In situ* N_2_O emission was also related to the abundance and proportion of *nosZII*, as well as to the total *nosZ* and *nosZ/nir* ratio in the spruce stands.

Contrary to N_2_O_10 *cm*_ emission, no prokaryotic genera were related to N_2_ emission from the 0–10 cm soils. However, like the *in situ* N_2_O emission, N_2_ emission was strongly related to the abundance and proportion of *CamoA* in soils where *CamoA* was detected (Supplementary Table 11). The proportions of *nifH, nirK*, and *nrfA* in the bacterial community of the birch stands, the B16S abundance, *AamoA* proportion, and *nosZI*/*nosZII* in the pine forests, and *nrfA* abundance and proportion of *nosZII* in total *nosZ* (*nosZII*/nosZ) in the spruce stands were related to N_2_ emission from the upper 10 cm soil layer (Supplementary Table 11).

Across all the forests, the value of the sink was positively related to the abundances of A16S, *nifH*, and *nirK*, and negatively related to *nirS* abundance and *nosZ/nir*.

**FIGURE 9:**
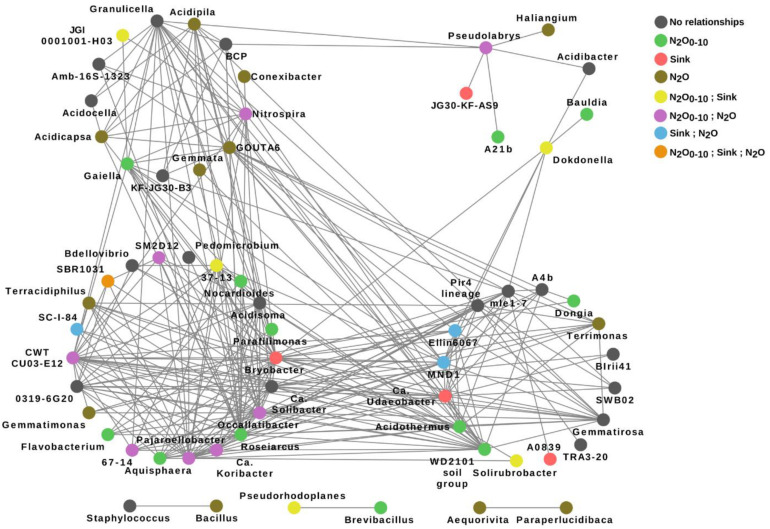
Microbial ecological network, based on SPIEC-EASI, showing relationships between prokaryotic genera in the soils of all studied forests. Differently colored nodes distinguish between genera that did not have significant relationships (according to RFR) and the genera significantly correlated with one or several measured gas parameters. Significant correlations were revealed with the N_2_O emission from 0 to 10 cm soil layer (N_2_O_0–10_) and sink in all forests, and with *in situ* N_2_O emission in the pine forests (N_2_O). Topological properties of the network: modularity 0.276, average connectivity 7.6, average clustering coefficient 0.50, degree 0.3, closeness 0.046, betweenness 0.11, and eigenvector 0.71.

Statistical analysis revealed that the content of TN and several of its forms were significantly related to the structure of some modules (SM1, SM4, and SM6) in the spruce microbial network (Supplementary Table 12). Network analysis based on prokaryotic genera proportions in soils of all the studied forests resulted in a network composed of a total of 58 prokaryotic genera that formed four bigger and three smaller modules (including 7–22 and two genera, respectively; Figure 9). Almost 65% of this network genera were related either to the *in situ* N_2_O emission, N_2_O_10 *cm*_ emission or sink, while the phylotype SBR1031 was correlated with all three parameters (Supplementary Table 12).

## Discussion

### Prokaryotic Community Structure in Soils of Different Forests

Despite similar abundances of bacteria in the soils of different forests, our results confirmed previous findings (Sun et al., 2014) that the dominant tree species has a major effect on the development of bacterial community structure in forests growing on drained peatlands. Congruent with other studies of forest soil microbial communities (Sun et al., 2014; Truu et al., 2017; Ren et al., 2019), the dominant bacterial phyla in the soils of this study were *Proteobacteria*, *Acidobacteria*, *Actinobacteria*, *Bacteroidetes*, *Planctomycetes*, and *Verrucomicrobia*. Significant differences between the bacterial communities, which were more pronounced between birch and pine forests, were revealed in terms of the proportions of both dominant and less abundant phyla and genera in the community. Communities of the spruce forests varied most between the stands and shared similarities with both the pine and the birch forest communities. Birch forest soils, which were the most N-rich, were distinguished from soils of the other studied forests by the higher proportions of *Bacteroidetes* – the specialists for the degradation of high molecular weight organic matter, such as proteins and carbohydrates (Thomas et al., 2011). The presence of the phyla *Chloroflexi*, *Nitrospirae*, and *Gemmatimonadetes*, which prefer N-rich environments (Sun et al., 2019; Li et al., 2020) were also a distinguishing feature of the birch forest soils.

The birch, pine, and spruce forests differed by both archaeal abundances and proportions in prokaryotic communities in soil; pine forest soils had the highest values while the birch forest soils had the lowest values in these aspects. Although *Thaumarchaeota* was the dominant archaeal group in most of the studied forests, the differences in archaeal community structure were more pronounced between the phylogenetically diverse pine stands and much less diverse birch forests. Again, the spruce forest stands shared similarities with both groups.

The relationships within a microbial community were less complex in the birch forest soils and only bacterial phylotypes appeared in the respective microbial network, while the pine forest network was the most complex. The only archaeal phylotype found in both coniferous forest networks belonged to the acidophilic class *Thermoplasmata* (phylum *Euryarchaeota*). Members of the class *Thermoplasmata* have previously been found in soils of boreal acidic forests (Kemnitz et al., 2007) and cold-temperate forests (Isoda et al., 2017), and are related to iron and sulfur cycling (Jin et al., 2016) and methanogenesis (Iino et al., 2013).

### The Effect of Soil Physicochemical Properties on Soil Prokaryotic Community

Chemical composition of the soil explained a large part of the microbial community variation. Soil chemistry (pH and composition of organic matter fractions) and tree species are strongly related (Sariyildiz et al., 2005; Berg, 2018) because the above- and below-ground litter, root exudates, and leachates vary according to the plant species (Quideau et al., 2001; Sariyildiz et al., 2005; Aneja et al., 2006; Merilä et al., 2010; Cakir and Makineci, 2013). Changes in soil physicochemical properties (soil texture, organic carbon, and TN content) and soil bacterial and fungal community structure at different stages (dominated by birch, pine or mixed communities) of the long-term development of natural forests have also been shown previously (Ren et al., 2019). In agreement with previous studies (Sariyildiz et al., 2005; Cakir and Makineci, 2013), the pine forest soils in this study were characterized by lower pH and dissolved nitrogen content. Pine litter contains significantly more lignin than the litter of deciduous trees (Sariyildiz et al., 2005; Cakir and Makineci, 2013). Furthermore, organic carbon sequestration rates are almost two-fold higher in coniferous forests compared with deciduous forests, while the rates in pine forests exceed those in spruce forests (Berg, 2018). Consequently, specific humus is accumulated in the forests dominated by different tree species (Kuiters, 1991; Sariyildiz et al., 2005; Berg, 2018) and the litter and humus quality (pH, lignin content and C/N ratio) affects the growth of microarthropods such as *Neelidae* and *Oribatid* (Cakir and Makineci, 2013), which are important degraders of organic matter and are related to microbial biomass in soil and litter (Chagnon et al., 2001).

Across all sites in this study, pH was the most significant factor affecting archaeal abundance and community structure and shaping the functional properties of both prokaryotic groups in soil. Archaeal abundance was highest in the most acidic pine forest soils. The effect of pH on soil bacterial community structure has been shown globally (Delgado-Baquerizo et al., 2018). Nitrogen status, total N content, and proportions of dissolved fractions, as well as the C/N ratio, also had a strong effect on the microbial community structure in the forest soils of this study. However, the pattern of relationships between the community characteristics and environmental factors differed between the three forest types.

A significant negative relationship between Ca concentration and archaeal abundance across all the sites was found, along with a strong effect of this element on the microbial community composition in the spruce forest. Calcium is known to promote lignin degradation by exerting a positive effect on lignin-degrading fungi (e.g., several *Marasmius* species) especially in acidic soils (Johansson, 1994; Berg et al., 1996). Active lignin degradation might represent a prerequisite for the formation of an abundant and diverse N-transforming community composed of comammox, AOB, and *nirS*-type complete denitrifiers, because the Ca concentration was positively related to the abundances of these organisms in soils analyzed in this study.

Previous studies have shown that specific plant communities, canopy cover, and average height of trees, different from the main forest area, develop in the vicinity (up to 400 m) of the ditches of transitional mires (Paal et al., 2016) and pine forests growing on the drained peatlands (Sisask, 2013). Spatial variation of carbon stock in the forest floor is affected by canopy structure through litterfall variation (Penne et al., 2010), which in turn might affect the development of soil microbial communities.

### The Effect of Tree Roots on Soil Prokaryotic Community

Tree fine root biomass (FRBt) and the ratio between tree and understory fine root biomass (FRBt/totFRB) were lowest in the pine forests, suggesting that the understory had a stronger impact on soil microbial community in these forests. Although the FRBt is similar in boreal and temperate forest ecosystems and is dependent on the density of trees (Finér et al., 2011), the proportions of both the absorptive and transport roots vary significantly along environmental gradients (Helmisaari et al., 2009; Ostonen et al., 2011).

The share of FRBt increased in the total FRB with increasing distance from the drainage ditch in the pine and birch forests and strongly affected the prokaryotic community structure of all the studied stands. Although this relationship was not evident in the birch forests of this study, probably because of the almost two-fold lower variation of the FRBt value in these soils relative to the coniferous forest soils, the complementarity of the bacterial community and absorptive roots (evaluated by morphological traits) to the changes in environmental conditions have previously been shown in silver birch forests (Truu et al., 2017). Furthermore, multilateral relationships between the absorptive roots, ectomycorrhizal fungi, and both soil bacterial communities (bulk soil and rhizosphere) across 10 silver birch stands indicated a significant role of each organism group, although the role of each associated partner was affected by environmental conditions such as the N and C/N status of the soil (Ostonen et al., 2017). In the current study, soil C/N and FRBt together explained a large part of the variation in the prokaryotic community structure of the pine forests. This corresponded with results of the earlier study that showed soil C/N ratio as the main factor causing variability in absorptive fine root biomass and its relations to the bacterial network modules structure in silver birch forests (Ostonen et al., 2017).

The strong relationship between the tree root biomass and prokaryotic community structure found across all the sites in this study refers primarily to the bacterial community dependence on the root exudates rather than on the dead root biomass in these forests. Through root exudate flux, the concentrations of metabolites may be locally enhanced in plants, thus regulating plant nutritional status. Nutrient availability in the surrounding environment is also influenced by plant-associated microorganisms as they regulate root exudation by increasing concentration gradients of metabolites in the soil (Canarini et al., 2019). It has been shown experimentally that ectomycorrhizal hyphae rapidly transfer plant-derived carbon to bacterial communities in beech root-distant areas and that this process promptly responds to changing nutrient conditions in the soil (Gorka et al., 2019).

### Nitrogen Transformation Potential in the Soils of Different Forests

The potential for almost all targeted N-transformation processes (except comammox) was detected in the soils of all the studied plots. The extent of the potential was often related to the conditions that developed in the soil following long-term growth of forest dominated by certain tree species. According to the results of RDA, the abundance of nine bacterial genera explained a large part of variation in the abundance of the studied N-transforming functional genes except for *nrfA*. Among these nine genera only *Occalatibacter* was differentially abundant between forest types. Analysis results indicate that variation in the abundance of *BamoA, nirS, nosZI*, and *nosZII* is related to the abundance of bacterial genera which strongly correlate with soil pH and to a lesser extent with fine root biomass. On the contrary, the variation in the abundance of *nifH* and *nirK* was associated with forest type. The bacterial genera related to RDA first axis formed very distinctive cluster with weak linkage to other modules in the community network.

The high proportion of *nifH* genes in the drained forest soils indicated that microbial N-fixation is a significant process for the acquisition of nitrogen by these ecosystems. Pine forests, characterized by FRBt that was two-fold lower than that of the other studied forests, had considerably higher numbers of N-fixers in the soils. This indicated that the root exudates and root litter were not sufficient N-providers in these forest soils and that N-fixation was essential for NH_3_ formation in soils characterized by low pH and high C/N ratio (low N concentration). Diazotrophy is widespread among the prokaryotic organisms (*Euryarchaeota* and 13 different bacterial phyla); however, not all organisms possessing some of the known *nif* genes are diazotrophs, because a set of genes encoding structural and biosynthetic components (*NifHDK* and *NifENB*) is necessary to perform this function (Dos Santos et al., 2012).

Dissimilatory nitrate reduction to ammonia organisms, a particularly important ammonia source in forest ecosystems (Paula et al., 2014), rely on the nitrite, the amount of which is dependent on N-fixation in soils with a low N pool such as the pine forest. In general, the abundance of DNRA organisms did not differ between the forest groups and was weakly related to microbial community structure; however, in the birch and spruce forests, it might be partly related to the presence of other microbial groups (e.g., *Ferruginibacter*).

Nitrification is a core process in the nitrogen cycle and ammonia oxidation is the first and rate-limiting step in this process (Lehtovirta-Morley, 2018). In this study, the proportion of different ammonia-oxidizing organisms was related to the soil pH and quality of organic matter (C/N ratio) – comammox bacteria preferred soils with the highest C/N ratio while AOB preferred soils with the lowest C/N ratio. AOB were much less abundant than N-fixers in the studied soils. A similar trend between the abundances of these organisms was observed in grassland soil during a vegetation period (Regan et al., 2017). The proportion of NOB exceeded that of well-known AOB (predominantly *Nitrosospira*) in the communities of the studied forest soils. However, the higher proportion of *Nitrospira* in communities of birch and spruce forests, and the significant number of *CamoA* genes found in the soils of these forests, indicates that comammox is an essential nitrate source in the birch and spruce forests. The analytical methods employed do not distinguish between individual species of the genus *Nitrospira* (Pjevac et al., 2017) or allow quantification of known comammox *amoA* clades separately (Wang et al., 2018). Wang et al. (2019) demonstrated that the diversity of comammox *Nitrospira* could be low and dominated by the clade B gene-possessing organisms in forest soils. In this study, there was a clear threshold value for soil pH (4.6) that defined the presence of comammox bacteria, and these organisms were not detected in the pine forest soils with a lower pH.

Ammonia oxidation kinetics (substrate affinity) has been shown to be a major driver of the niche separation between AOB and archaea and comammox bacteria (Kits et al., 2017). The comammox bacteria have been suggested to be advantageous especially in nutrient-limited environments (Koch et al., 2019). Although the ammonia content was often low (<1.5 mgN kg^–1^ dw^–1^), there was no correlation between the ammonia concentration and *CamoA* gene abundance in soils of this study.

The predominant archaeal phylum, *Thaumarchaeota*, was shown to be the most abundant and ubiquitous group of microorganisms in soils and contain besides ammonia-oxidizing organisms (Group1.1a and Group 1.1b), also groups (Group 1.1c) that have not been associated with ammonia oxidation previously (Weber et al., 2015). Genus Ca. *Nitrosopumilus* from the Group1.1a, was significantly less abundant in spruce forest soils than in those of the other two forest groups and correlated positively with *AamoA* abundance. In addition to ammonia oxidation, this genus contains organisms with the potential to perform several other N-transforming processes including denitrification (e.g., *N. adriaticus* possessing *nirK*, *N. piranensis* possessing *nirS*, and *N. sediminis* sp. AR2 possessing *nirS* and *nosZ*), and reduction of other nitrogen-containing compounds by producing nitroreductases (*N. adriaticus*) (NCBI). Although the final RDA model did not contain any archaeal taxa, the inclusion of the Ca. *Nitrosopumilus* to RDA plot as a passive variable indicated a positive correlation of this genus with abundance of several N-transforming gene abundances.

Similar to some other forest soils (Truu et al., 2017), the soils of drained peatland forests favored *nirK*-type denitrifiers that dominated over the *nirS*-type organisms. However, the abundance and proportion of *nirK*-type organisms in the soils of coniferous forests considerably exceeded those of birch forest soils. According to RDA, the genus *Occallatibacter* has a strong positive relationship with *nirK*-type denitrifiers. Known *Occallatibacter* strains are aerobic, chemoheterotrophic mesophiles with a broad temperature range for growth and a moderately acidic pH optimum (Foesel et al., 2016) and *Occallatibacter savannae* genome harbors *nirK* gene (NCBI).

The *nosZ/nir* ratio in the studied soils indicated that the abundance of complete denitrifying organisms was significantly lower in the pine forests, while in the birch forests, this ratio was highest and most variable. As with the arable soils, lake sediments, and other environments analyzed by Jones et al. (2013), both clades of *nosZ* gene were represented in the drained peatland forest soils, and the abundance of clade II organisms even slightly exceeded the abundance of clade I organisms. The clades were not divided by the forest groups and, contrary to some other studies (Ligi et al., 2015), similar environmental factors (especially pH, nitrate, and Ca content) affected the abundance of these groups in peatland forests. Bacterial genera related to the abundances of both *nirS* and *nosZ* (e.g., *Haliangium*, A21b, *Pseudolabrys*) were almost equally represented in soil communities of different forests types. The proportion of the genus *Pseudolabrys*, whose members metabolize organic acids (Kämpfer et al., 2006) and increase in response to N addition to soil (Eo and Park, 2016), was significantly higher in the birch forest soil communities than in those of the other two forest types and was related to the *nirS* and *nosZII* abundances in this study. Contrary to the silver birch stands grown on former agricultural land (Truu et al., 2017), the results of this study suggest that there were more complete denitrifiers among the *nirS*-type denitrifiers because the demands for environmental conditions (pattern of significant factors) of these organisms more closely resembled those of *nosZ*-possessing organisms in the studied soils.

Tree roots had a substantial effect on N-cycling microbes. In general, higher numbers of AOB (both canonical and comammox) and the abundance and proportion of *nirS*-type denitrifiers in the community of denitrifying organisms corresponded to forests with a larger FRBt and probably a higher number of root tips per soil volume. A previous study on bulk soil bacterial communities of silver birch stands found a relationship between the root branching intensity, which increased the input of labile root exudates into the soil, and the proportions of *nirS*-type denitrifiers, as well as the *nir/nosZ* ratio (Truu et al., 2017).

### Effect of Drainage Ditches on N-Cycling Organisms and N-Gas Emissions

Previous studies have shown that the water table controls greenhouse gas emissions as well as available N and P pools in soil, and also regulates the C/N ratio of vascular plants in boreal forested peatland (Munir et al., 2017; Laine et al., 2019). There were no observed trends in soil chemistry and water table across the distance gradients at the time of sampling in this study; however, the effect of the drainage ditch on the spatial distribution of the N-cycling microbes was evident. This effect was coupled with the characteristics for each particular stand, although often the effect of forest type was also involved.

The results indicate that besides the aforementioned plant community changes and concordant effects on soil chemistry and physics (light intensity, temperature, etc.), the water conditions affected the spatial distribution of soil microbes in the drained peatland forest soils. Although the water table was below the peat layer at the time of sampling, the effect of the drainage was stronger in the vicinity of the ditches where larger fluctuations in SWC occurred. The drainage effect on different functional groups varied in different forest groups. The abundances of N-fixing organisms increased with increasing distance from the drainage ditch across all the stands and were significantly related to the SWC. Often, systematic changes in abundance of these groups occurred in the plots up to a distance of 40 m from the drainage ditch, while the 80 m plots (especially in spruce forests) differed in their community structure and functional ability. For instance, the abundances of ammonia-oxidizing organisms (both AOB and AOA) were related to the SWC, and clustering of the plots according to their nitrifying community structure and abundance by forest type and location was evident. The community structure (*nirS*/*nirK* and *nozZI*/*nosZII*) and abundance of denitrifiers were dependent on the plot distance from the ditch. The dependence of *nirS*-type organisms on SWC has previously been demonstrated (Regan et al., 2017).

*In situ* N_2_O emissions from the studied forests were considerably lower than those recorded for other boreal peatland forests with a similar drainage history or undrained boreal peatlands (Laine et al., 2019), and were more similar to values from a northern peatland forest and drained peatland reported by Liimatainen et al. (2018). In general, higher *in situ* N_2_O emissions were recorded from the soils that had a phylogenetically more diverse prokaryotic community, although consumption of N_2_O in the upper 10 cm soil layer was prevalent in most plots at the time of sampling. N_2_O emission and sink were dependent on N-fixation and the proportion of complete denitrifying organisms (possessing *nosZ* clade I) in the community of pine forest soils, while *nirK*-type denitrifiers were responsible for N_2_O emission and sink in the upper layer of birch forest soils. Some N_2_O may originate from *nirK*-possessing fungi as they are significant producers of N_2_O in terrestrial environments, including forest ecosystems (Maeda et al., 2015). The abundance of nitrifying bacteria (both AOB and comammox) and the proportion of DNRA organisms in the community were positively related to N_2_O reduction in the studied soils, and the link between N_2_ emission and DNRA was especially pronounced in the birch and spruce forest soils.

Drainage also affected N_2_ production because the abundance of N_2_O reducers was strongly dependent on the water content of peat. Fluctuating moisture conditions were shown to affect nitrification less but significantly increase denitrification rates in upland and wetland soils (Peralta et al., 2013).

## Conclusion

This study indicated that long-term growth of forests dominated by birch, pine, and spruce on initially similar organic soil has caused significant, tree-species-specific changes in the soil properties, which has led to the development of forest type-specific soil prokaryotic communities with characteristic functional properties and relationships between the organisms. Our study demonstrated that, in general, soils with lower tree root biomasses and turnover rates, i.e., pine forest soils, had higher archaeal abundances. The positive relationship between the C/N ratio and archaeal abundance implies that these organisms are dependent on recalcitrant organic material deposited in the bulk soil of drained peatland forests. The abundances of N-fixers and *nirK*-type denitrifiers were more strongly related to the effect of dominant tree species than the abundances of *nirS*-type denitrifires, N_2_O reducers and nitrifiers. The comammox organisms were abundant in the drained peatland forests with soil pH higher than 4.6. The drainage ditches affected the microbial community structure and functions; these effects were strongly dependent on both the dominant tree species and the specific site conditions, such as the thickness and chemical composition of the peat layer, and the ratio of tree and understory biomass. The N_2_O emission was higher from soils that had higher microbial community phylogenetic diversity and the N_2_O consumption was recorded in the upper soil layer in most cases. Depending on the dominant tree species, the different microbial functional groups were related to the N_2_O emission from soil.

## Data Availability Statement

The datasets presented in this study can be found in online repositories. The names of the repository/repositories and accession number(s) can be found below: https://ebi.ac.uk/ena, PRJEB38904.

## Author Contributions

MT, IO, VU, ÜM, MM, and JT designed the study. MT, IO, ME, and MM performed the fieldwork and collected the data. MT, HN, TL, IO, and MM performed laboratory analysis. KO, JT, and ME conducted the bioinformatics and statistical analyses. MT wrote the first draft of the manuscript. All authors contributed to the manuscript revisions and improvements until the submitted version.

## Conflict of Interest

The authors declare that the research was conducted in the absence of any commercial or financial relationships that could be construed as a potential conflict of interest.
