# Supplementary material for: Soil Bacterial and Archaeal Communities and Their Potential to Perform N-Cycling Processes in Soils of Boreal Forests Growing on Well-Drained Peat
Source: Front Microbiol. 2020 Dec 3;11:591358. doi: 10.3389/fmicb.2020.591358 (PMC7744593; doi:10.3389/fmicb.2020.591358)
Supplement: Supplementary file 1 [file Data_Sheet_1.docx]

Supplementary Material

# Supplementary Data

## Soil analyses

Soil acidity (pH_H20_) and total carbon (TC), nitrogen (TN), phosphorus (TP), potassium (K) and sulfur (S) contents, as well as water-soluble ammonium and nitrate nitrogen (NH_4_-N and NO_3_-N, respectively), phosphate phosphorus (PO_4_-P) and calcium ion (Ca^2+^) concentrations, were measured with the standard methods in the laboratory of the Estonian Environmental Research Centre. Additionally, the concentrations of dissolved organic carbon (DOC) and dissolved nitrogen (DN) were measured from soil using Vario TOC equipped with a TNb detector (Elementar GmbH, Germany). All the chemical parameter values were presented on the basis of dry soil.

## Plant root sampling and analysis

Fine root (diameter <2 mm) biomass (g m^-2^) of trees (FRBt) and understory (FRBu) was measured by soil cores (ø 38 mm). In each stand, 20 cores (5 per each sampling plot) were randomly collected from the top 10 cm at the end of the growing season. The proportion of fine root biomass of trees from the total plant fine root biomass (FRBt/totFRB) was calculated for all subplots (n=5). For estimating fine root production and turnover, fine root ingrowth nets (Hirano et al., 2009) were installed to all stands and plots in autumn of 2013 and 2014 and extracted in 2014-2016. The turnover rate (TR, yr^-1^) of tree fine roots was calculated from nets (Kriiska et al., 2019) and applied in the calculation of fine root production (FRP, g m^2^ yr^-1^).

## Gas and soil sampling for the measurement of gas fluxes

Four collars (2m apart) for the static closed chambers (Ø 50 cm, volume 65 L) for *in situ* measurements of gas emissions from soil surface were placed on each plot of all study sites. The gas emissions from the whole soil core, as well as the groundwater level, temperature, and other soil parameters, were monitored with a three-week interval.

Nitrous oxide (N_2_O) emission was measured *in situ* using static closed chambers (Ø 50 cm, height 40 cm, and volume 65 L) sealed with a water-filled collar on the soil surface.

The gas samples were taken at the beginning, after 20, 40 and 60 minutes from the enclosure of samplers using pre-evacuated (0.3 mbar) 50-mL gas bottles. The targeted gas concentration in the collected air was determined using the Shimadzu GC-2014 gas-chromatographic system equipped with an electron capture detector (ECD) and a flame ionisation detector (FID) and combined with a Loftfield autosampler (Loftfield et al., 1997). The means of N_2_O and CO_2_ emissions obtained during 10 sampling sessions from May to November 2014 (in total 40 measurements per plot) were used to evaluate the relationships between gas emissions to the soil, plants and microbiological parameters in further data analyses.

During the soil sampling session, an intact soil core from the 0-10 cm soil layer of each sampling plot was collected into stainless steel cylinder (Ø 6.8 cm) for the analysis of gas emissions (N_2_, and N_2_O) in the laboratory. The cylinders were air-tightly sealed and kept at cool conditions (4 °C) until the helium atmosphere soil incubation (Swerts et al., 1995; Butterbach-Bahl et al., 2002) in the laboratory. The cylinders with intact soil cores were placed into special gas-tight incubation vessels locating in a climate chamber. The gases were removed from the cylinders by flushing the soil cores with an artificial gas mixture (21.0% O_2_, 358 ppm CO_2_, 0.313 ppm N_2_O, 1.67 ppm CH_4_, 5.97 ppm N_2_ and rest He). The new atmosphere equilibrium by continuously flushing the vessel headspace with the artificial gas mixture at 20 mL per min was established after 12–24 h. The flushing time depended on the soil moisture. The temperature was kept similar to the field conditions during the incubation. A gas-chromatograph (Shimadzu GC-2014) equipped with a thermal conductivity detector was used to measure N_2_ concentration in the mixture of emitted gases accumulated in the headspaces (start value, 40 min, 80 min, and 120 min as final value) of the cylinders after 2 h of closure. The gas concentration in the chambers increased in a near-linear fashion and linear regression was applied and the flux measurements with R^2^ of 0.81 (p<0.1) or greater were used for the calculations of gas fluxes (Butterbach-Bahl et al., 2002). From the gas emissions measured in the laboratory, also a sink (N_2_O/(N_2_O+N_2_)) was calculated for the 0-10 layer of soil of each sampling plot.

## PCR products preparation and sequencing

All PCR reactions were performed in a 20 μL reaction mixture using a Phusion Hot Start High Fidelity Polymerase (Thermo Fisher Scientific, Waltham, MA) according to the manufacturer’s instructions. The DNA template concentration in the reaction mixture varied between 0.9–1.0 ng/μl. The amplification of each sample was performed in triplicate with the following PCR program: initial denaturation at 98 °C (30 s) followed by 25 cycles of denaturation at 98 °C (10 s), annealing at 60 °C (30 s), extension at 72 °C (15 s) and final extension step at 72 °C for 8 min. The replicate PCR products were pooled, and the concentration of each composite sample was determined with the TapeStation 2200 using D1000 ScreenTapes ® (Agilent Technologies, Inc., Santa Clara, CA). Amplicons of all samples were finally pooled in equal proportions and the mixture was purified and concentrated using the NucleoSpin® Extract II kit (MACHEREY-NAGEL GmbH & Co. KG, Düren, Germany). The paired-end DNA library was prepared according to Herbold and co-authors (Herbold et al., 2015) and sequenced on an Illumina® MiSeq system (Illumina Inc., San Diego, USA) at Microsynth AG (Balgach, Switzerland).

## Sequence data preparation and taxonomic assessment

The paired-end reads were assembled into composite reads with Pear v 0.9.11 (Zhang et al., 2014). Bacterial and archaeal sequences were separated by adapter tags using BBMap v 37.86 (https://sourceforge.net/projects/bbmap/). The assembled reads were processed using Mothur v 1.40.4 (Schloss et al., 2009). Illumina reads were demultiplexed and during data denoising, sequences were discarded if the average sequencing quality score dropped below 25 over a 25-bp sliding window, were shorter than 100 bp, had any ambiguous bases, and had longer than 6 homopolymers. Chimeras were detected with vsearch algorithm in de novo mode (Rognes et al., 2016). SILVA alignment v 132 (Pruesse et al., 2007) was used as a reference database for taxonomic assignment. 80% confidence in bootstrap values was used for classifying sequences. Eukaryotes and reads that were taxonomically unable to assign were removed from the following analysis. Quality checked reads were clustered into operational taxonomic units (OTUs) using distance-based greedy clustering method with a 95% similarity threshold (Westcott and Schloss, 2015). Spurious OTUs with less than 3 sequences were discarded.

## Quantitative PCR conditions and data analysis

The qPCR assays were performed using RotorGene® Q (Qiagen, Valencia, CA). Stock solutions of target sequence containing plasmids (Eurofins MWG Operon, Ebersberg, Germany) were used to create serially diluted standard curves. The qPCR reactions were performed in 10 μL volume containing 5 μL Maxima SYBR Green Master Mix (Thermo Fisher Scientific Inc.), optimised concentrations of forward and reverse primers, 1 μL of template DNA and sterile distilled water. The gene-specific primer sets, optimised primers concentrations, thermal cycling conditions, standard curve ranges and quantification limits (LOQ) for each target gene are shown in Table S1. All qPCR measurements were performed in triplicates.

The quantification data were analysed with RotorGene Series Software v.2.0.2 (Qiagen) and LinRegPCR program v.2017.0 (Ruijter et al., 2009). The gene abundances were calculated as a mean of fold differences between a sample and each 10-fold standard dilution in respective standard as proposed by Ruijter et al., (2009) and presented as gene copy numbers per gram of dry soil (copies g^-1^ dw^-1^). The proportion of archaea in the total prokaryotic organisms’ community was calculated. Additionally, the proportions of the genes of the functional groups in archaeal and bacterial communities were estimated by normalizing the functional gene abundances with the 16S rRNA genes of the respective host organism group.

The ratios between two types of *nir* genes (*nirS*/*nirK*), two *nosZ* gene clades (*nosZI*/*nosZII*) as well as ratios between total *nir* (*nirS*+*nirK*) and *nosZ* (*nosZI*+*nosZII*) genes (*nir*/*nosZ*) were also calculated.

# Supplementary Figures and Tables

## Supplementary Figures


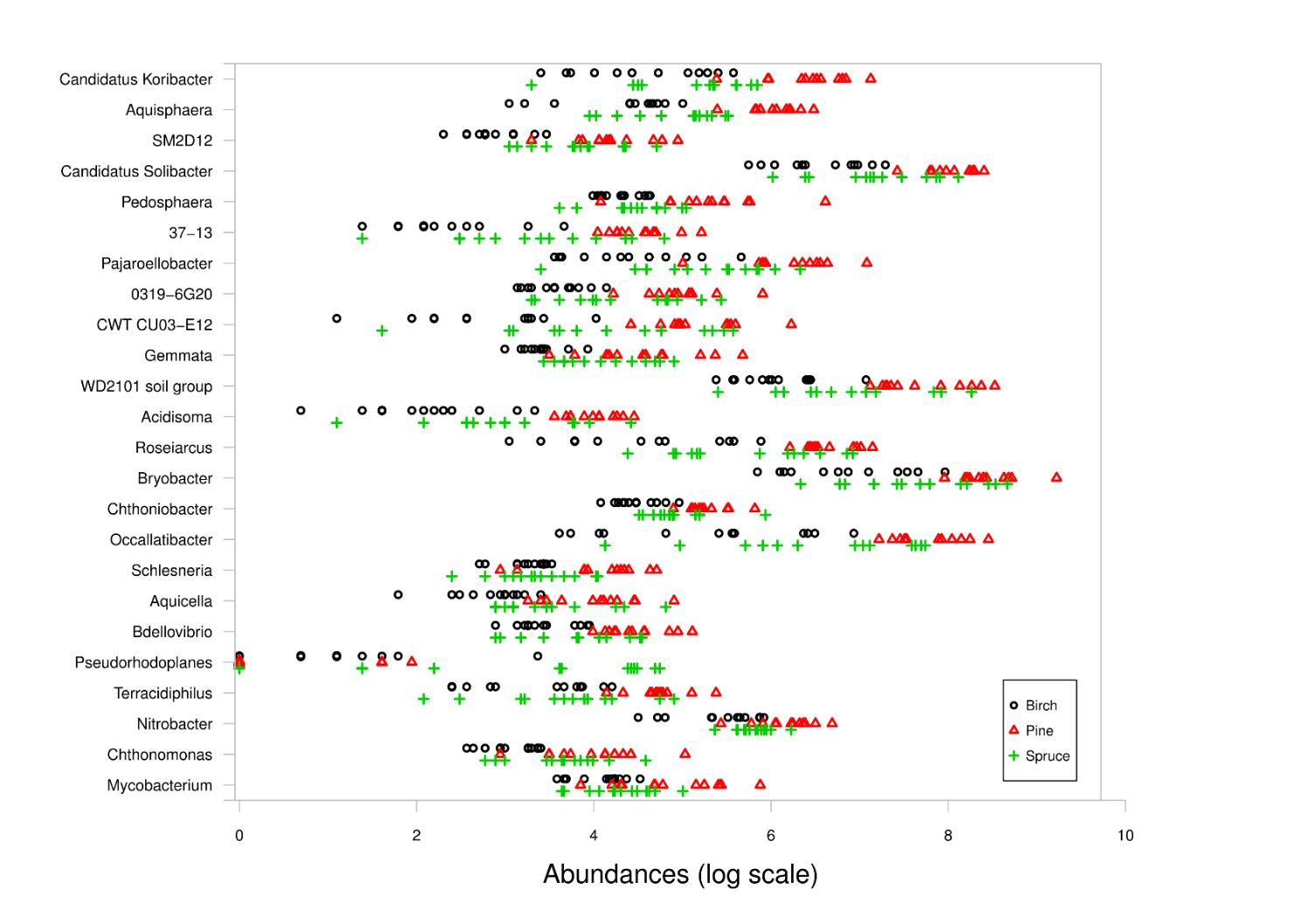


**Supplementary Figure 1.** The plot of bacterial genera that were found to be different between forest types (MVANOVA, p<0.01 up to the *Roseiarcus* and p<0.05 from *Bryobacter*) in the studied forests. Transformed ($log(\frac{y}{min}+1))$bacterial genera abundance values are shown on X-axis.


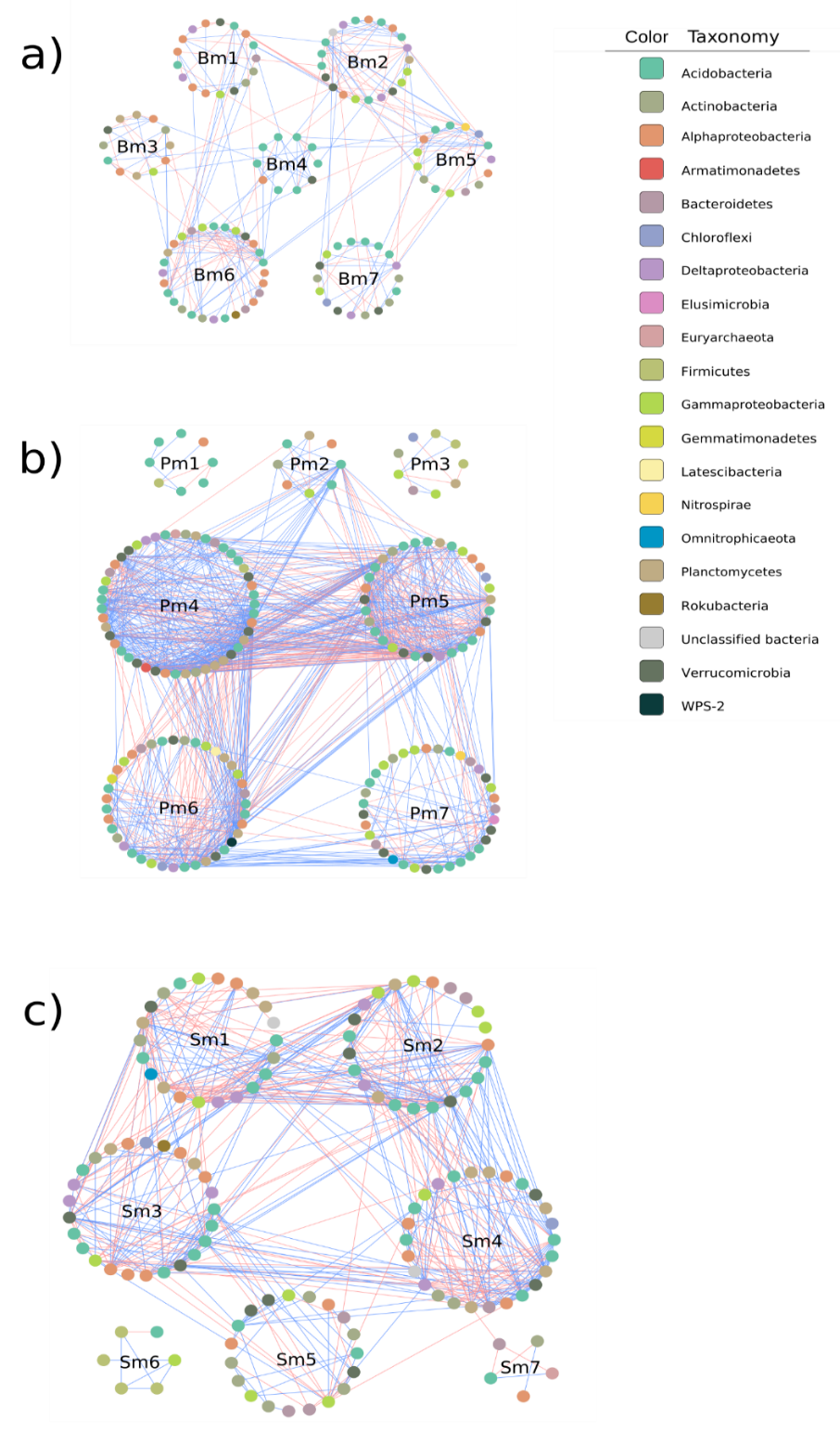


**Supplementary Figure 2.** The microbial ecological networks (based on SPIEC-EASI) obtained from OTU data for the birch (a), pine (b) and spruce (c) forests. The nodes are colored according to the OTUs phyla and the modules (m) of each forest type network have numeration from 1 to 7. The lines connecting OTUs within a module and between the modules show the edge stability of the associations based on conditional independence.


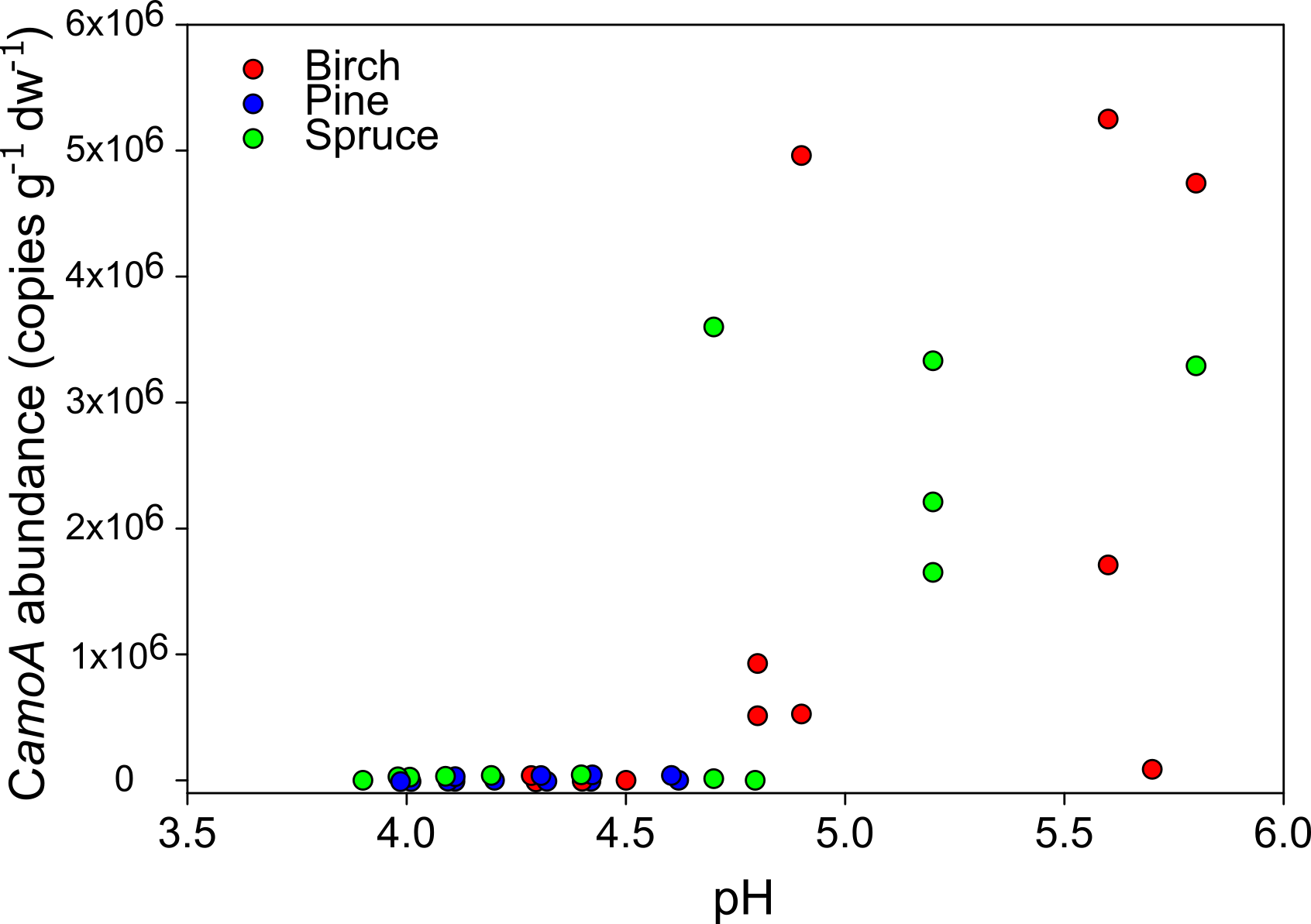


**Supplementary Figure 3.** Scatterplot showing relationships between soil pH and comammox-specific *amoA* abundance in the studied forest soils.


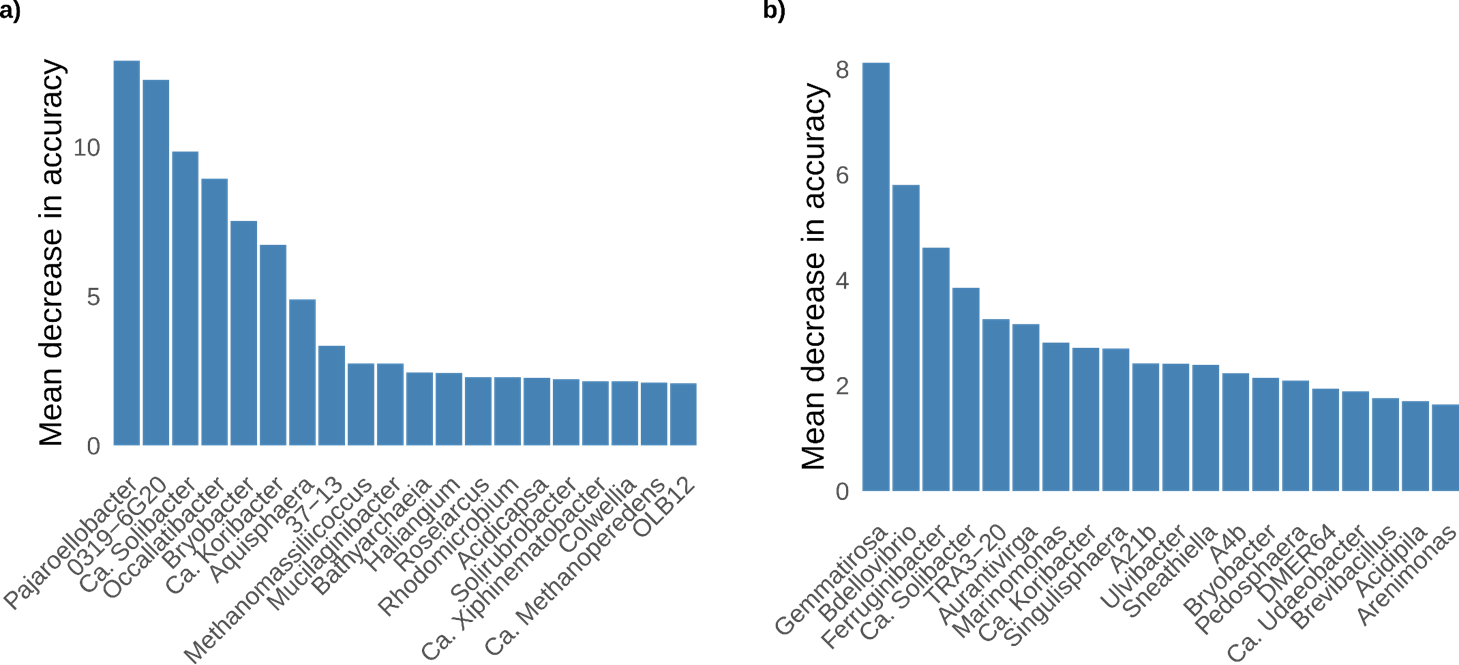


**Supplementary Figure 4.** Top 20 genera (out of 35 and 29 genera) that estimated abundances were significantly related (according to RFR) to the variation in the *nifH* (a) and *nrfA* (b) gene abundances (47.4% and 29.3% of the variation explained, respectively) in the soils of studied forests.


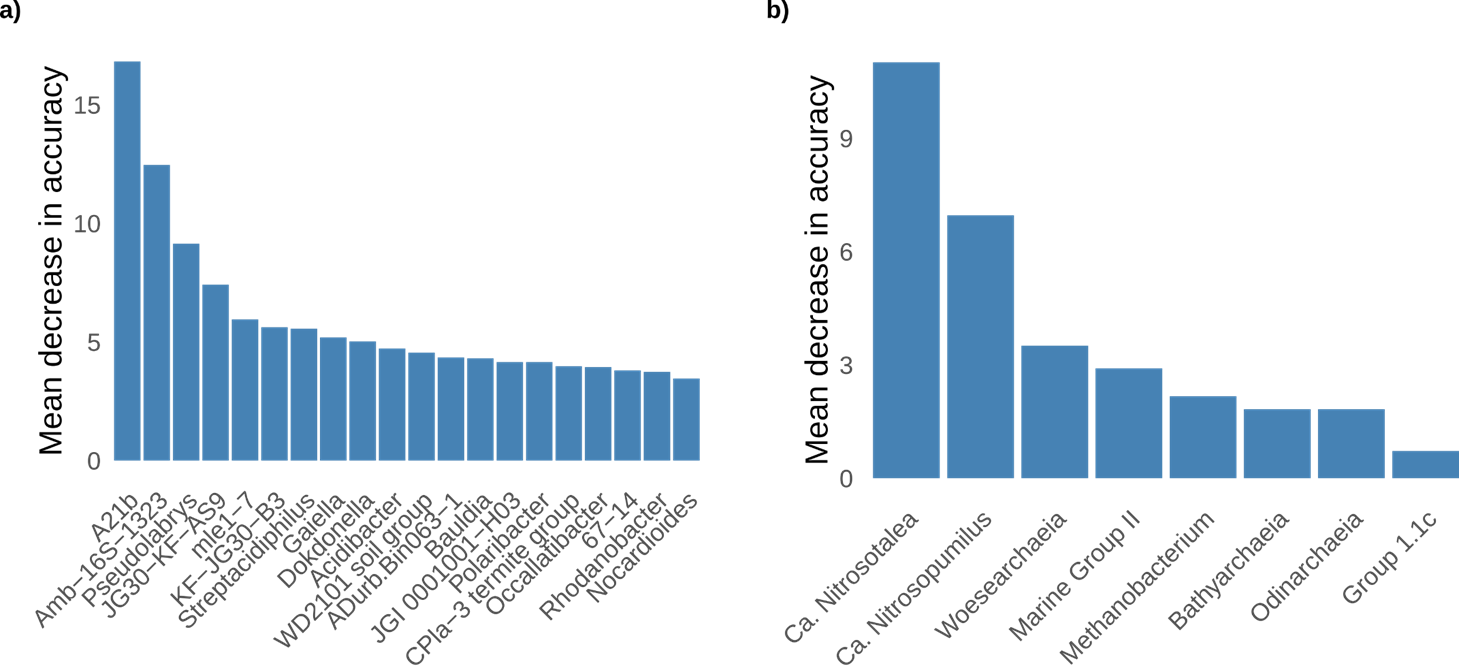


**Supplementary Figure 5.** Top 20 genera (out of 40 genera) that estimated abundances were significantly related (according to RFR) to the variation in the *BamoA* (a) gene abundance (65.5% of the variation explained) and all the archaeal genera (b) that estimated abundances explained 68.7% of the *AamoA* gene abundance variation in the soils of studied forests.


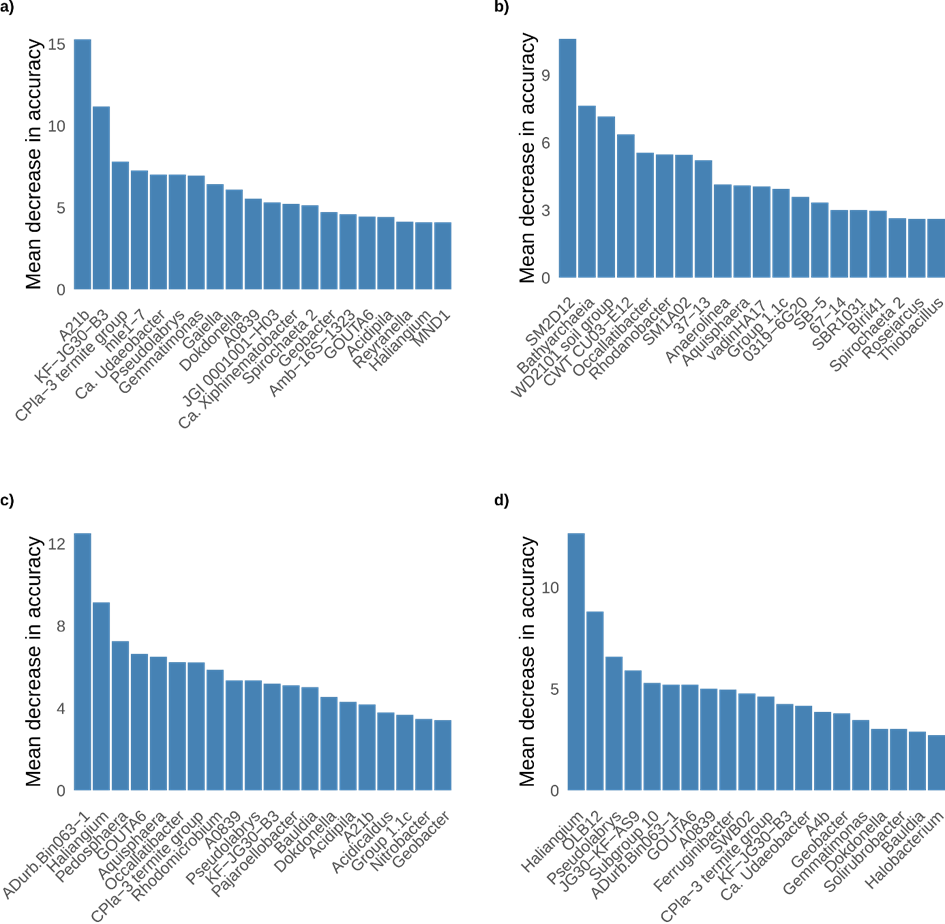


**Supplementary Figure 6.** Top 20 genera (out of 70, 57, 63 and 58 genera) that estimated abundances were significantly related (according to RFR) to the variation in the *nirS* (a), *nirK* (b), *nosZI* (c) and *nosZII* (d) gene abundances (89.2%, 43.3%, 55.4% and 49.9% of the variation explained, respectively) in the soils of studied forests.


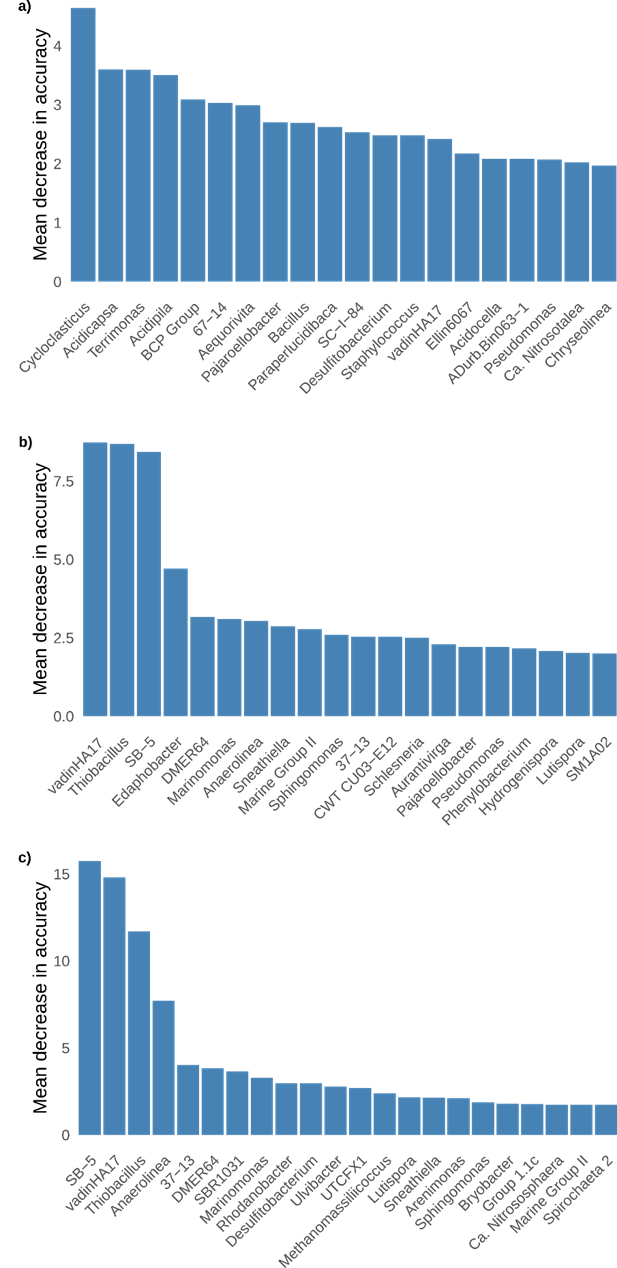


**Supplementary Figure 7.** Top 20 prokaryotic genera (out of 62, 40 and 54) that estimated abundances were significantly related (according to RFR) to the in situ N_2_O emission variation (a), as well as the top 20 genera related to the N_2_O emission from 0-10 cm soil layer (b) and sink in 0-10 cm soil (c) in all the studied soils (53.1%, 24.8.0%, and 60.0% of the variation explained, respectively).

## Supplementary Tables

**Supplementary Table 1.** Characteristics of qPCR primer pairs and programs. LOQ, limit of quantification.

| **Target gene** | **Primers** | **Primer sequence 5´-3´** | **Amplicon size (bp)** | **Primer conc (µM)** | **qPCR program** | **qPCR assay LOQ (copies in reaction)** | **Primer reference** |
| --- | --- | --- | --- | --- | --- | --- | --- |
| Bacterial 16S rRNA | Bact517F | GCCAGCAGCCGCGGTAA | 530 | 0.6 | 50°C 2 min, 95°C 10 min; 35 cycles: 95°C 30 s; 60°C 45 s; 72°C 45 s | 1000 | Liu et al., 2007 |
|  | Bact1028R | CGACARCCATGCASCACCT |  | 0.6 |  |  | Dethlefsen et al., 2008 |
| Archaeal 16S rRNA | Arc519F | CAGYCGCCRCGGTAA | 393 | 0.6 | 50°C 2 min, 95°C 10 min; 35 cycles: 95°C 15 s; 56°C 30 s; 72°C 30 s | 1000 | Espenberg et al., 2016 |
|  | Arch910R | GAATWGGCGGGGGRGC |  | 0.6 |  |  |  |
| Bacterial *amoA* | amoA-1F | GGGGTTTCTACTGGTGGT | 491 | 0.8 | 50°C 2 min, 95°C 10 min; 35 cycles: 95°C 30 s; 60°C 45 s; 72°C 45 s | 25 | Rotthauwe et al., 1997 |
|  | amoA-2R | CCCCTCKGSAAAGCCTTCTTC |  | 0.8 |  |  |  |
| Archaeal *amoA* | CrenamoA 23F | ATGGTCTGGCTWAGACG | 600 | 0.8  0.8 | 50°C 2 min, 95°C 10 min; 35 cycles: 95°C 30 s; 55°C 45 s; 72°C 45 s | 50 | Tourna et al., 2008 |
|  | CrenamoA 616R | GCCATCCATCTGTATGTCCA |  |  |  |  |  |
| Comammox *amoA** | comamoA AF | AGGNGAYTGGGAYTTCTGG | 436 | 0.8 | 50°C 2 min, 95°C 10 min; 35 cycles: 95°C 15 s; 55°C 30 s; 72°C 30 s |  | Wang et al., 2018 |
|  | comamoA SR | CCGVACATACATRAAGCCCAT |  | 0.8 |  |  |  |
| *nrfA* | 6F | GAYTGCCAYATGCCRAAAGT | 222 | 0.8 | 50°C 2 min, 95°C 10 min; 45 cycles: 95°C 15 s; 55°C 30 s; 72°C 30 s | 25 | Takeuchi, 2006 |
|  | 6R | GCBKCTTTYGCTTCRAAGTG |  | 0.8 |  |  |  |
| *nifH* | Ueda19F | GCIWTYTAYGGIAARGGIGG | 390 | 0.8  0.8 | 50°C 2 min, 95°C 10 min; 40 cycles: 95°C 30 s; 53°C 45 s; 72°C 45 s | 100 | Ueda et al., 1995 |
|  | Ueda407R | AAICCRCCRCAIACIACRTC |  |  |  |  |  |
| *nirS* | nirSC1F | ATCGTCAACGTCAARGARACVGG | 431 | 0.8 | 50°C 2 min, 95°C 10 min; 40 cycles: 95°C 15 s; 58°C 30 s; 72°C 30 s; 80°C 30 s^a^ | 25 | Wei et al., 2015 |
|  | nirSR3cd | GASTTCGGRTGSGTCTTSATGAA |  | 0.8 |  |  | Kandeler et al., 2006 |
| *nirK* | nirK876 | ATYGGCGGVCAYGGCGA | 165 | 0.8 | 50°C 2 min, 95°C 10 min; 40 cycles: 95°C 15 s; 58°C 30 s; 72°C 30 s; 80°30 s^a^ | 100 | Henry et al., 2004 |
|  | nirK1040 | GCCTCGATCAGRTTRTGGTT |  | 0.8 |  |  |  |
| *nosZI* | nosZ2F | CGCRACGGCAASAAGGTSMSSGT | 267 | 0.8 | 50°C 2 min, 95°C 10 min; 40 cycles: 95°C 15 s; 60°C 30 s; 72°C 30 s; 80°C 30 s^a^ | 50 | Henry et al., 2006 |
|  | nosZ2R | CAKRTGCAKSGCRTGGCAGAA |  | 0.8 |  |  |  |
| *nosZII* | nosZIIF | CTIGGICCIYTKCAYAC | ~700 | 0.8 | 50°C 2 min, 95°C 10 min; 45 cycles: 95°C 30 s; 54°C 45 s; 72°C 45 s; 80°C 45 s^a^ | 10 | Jones et al., 2013 |
|  | nosZIIR | GCIGARCARAAITCBGTRC |  | 0.8 |  |  |  |

### ^a^ Fluorescence signal was read after the second extension step (80 ˚C)

### *Specificity of all obtained products was confirmed by D1000 ScreenTapes assay using 2200 Tape Station system (Agilent Technologies)

**Supplementary Table 2**. The means and standard deviations of the soil chemical characteristics of the studied stands. B 1-3 – birch stands; S 1-3 – spruce stands; P 1-3 pine stands; TC - total carbon, DOC – dissolved organic carbon, TN – total nitrogen; DN – dissolved nitrogen; TP – total phosphorous. All the concentrations (except the TC) are expressed on the basis of dry soil.

|  | Stands | | | | | | | | |
| --- | --- | --- | --- | --- | --- | --- | --- | --- | --- |
| Parameter | Downy birch | | | Norway spruce | | | Scots pine | | |
|  | B1  (n=4) | B2  (n=4) | B3  (n=4) | S1  (n=4) | S2  (n=4) | S3  (n=5) | P1  (n=4) | P2  (n=4) | P3  (n=4) |
| pH_H2O_ | 4.38±0.10 | 5.68±0.10 | 4.85±0.06 | 5.08±0.25 | 4.93±0.61 | 4.04±0.11 | 4.08±0.05 | 4.48±0.15 | 4.23±0.17 |
| TC (%) | 50.8±0.5 | 45.3±1.0 | 48.5±1.3 | 47.8±0.5 | 42.3±11.0 | 49.2±1.6 | 51.0±1.2 | 48.3±2.5 | 50.5±0.6 |
| DOC (gC/kg) | 1.2±0.1 | 1.1±0.2 | 1.8±0.3 | 1.7±0.5 | 1.4±0.4 | 1.8±0.2 | 1.4±0.1 | 1.5±0.5 | 1.7±0.4 |
| TN (g/kg) | 23.8± 1.0 | 28.0±1.0 | 25.8±7.2 | 25.5±1.3 | 19.0±3.2 | 19.4±1.1 | 15.3±1.0 | 20.0±2.6 | 22.3±1.5 |
| DN (mg/kg) | 71±16 | 111±12 | 188±17 | 124±27 | 66±14 | 63±16 | 46±6 | 69±21 | 89±24 |
| NH_4_-N (mg/kg) | 9.45±2.92 | 0.00±0.00 | 20.3±3.9 | 3.95±3.84 | 1.95±3.90 | 0.00±0.00 | 0.00±0.00 | 2.8±2.0 | 9.53±5.91 |
| NO_3_-N (mg/kg) | 6.10±3.67 | 57.5±14.7 | 70.5±19.8 | 38.8±22.1 | 4.95±3.64 | 4.62±1.56 | 2.78±0.74 | 8.30±6.14 | 13.0±7.8 |
| C/N | 50.8±0.5 | 16.2±0.7 | 20.6±8.5 | 18.8±1.0 | 22.0±3.8 | 25.5±2.0 | 33.5±0.9 | 24.3±2.1 | 22.8±1.5 |
| TP (g/kg) | 0.95±0.07 | 2.48±0.13 | 1.25±0.38 | 1.15±0.06 | 0.71±0.04 | 0.83±0.09 | 0.74±0.06 | 0.92±0.09 | 1.07±0.07 |
| PO_4_-P (mg/kg) | 0.40±0.05 | 2.55±0.95 | 1.41±0.67 | 0.36±0.25 | 0.39±0.49 | 0.86±0.91 | 0.65±0.25 | 0.41±0.21 | 0.54±0.46 |
| S (g/kg) | 2.2±0.1 | 2.7±0.2 | 2.5±0.6 | 2.5±0.2 | 1.9±0.1 | 2.0±0.2 | 1.6±0.1 | 2.1±0.1 | 2.5±0.1 |
| SO_4_ (mg/kg) | 72±19 | 59±12 | 103±21 | 82±41 | <53 | <53 | <53 | 58±12 | 57±11 |
| K (mg/kg) | 523±13 | 435±37 | 555±49 | 485±60 | 648±147 | 552±161 | 718±76 | 428±34 | 455±44 |
| Ca^2+^ (mg/kg) | 73±32 | 208±12 | 240±27 | 229±28 | 146±59 | 61±20 | 22±10 | 72±17 | 69±9 |

**Supplementary Table 3**. Means and standard deviations of the gas emissions measured from the 0-10 cm layer of the birch (B), pine (P) and spruce (S) forest at the laboratory conditions (n= 3) and emissions from the whole soil column (*in situ* emissions, n=160) during the growing season (from April to November 2014), as well as the following plant traits: fine root biomass of trees (FRBt), proportion of the FRBt in the total fine root biomass (FRBt/totFTB, where the totFRB is a sum of FRBt and understory FRB), and fine root turnover rate (TR) per year (n=3). The number at the plot code subscript shows the plot distance (in meters) from the drainage ditch.

| Plots | Gas emission from 0-10 cm layer | | *In situ* gas emission | Plant traits | | |
| --- | --- | --- | --- | --- | --- | --- |
|  | N_2_  (mg-N/m h) | N_2_O  (µg-N/m h) | N_2_O  (µg-N/m h) | FRB  (g/m^2^) | FRBt/totFRB | TR |
| B_5_ | 0.66±0.44 | -5.25±1.43 | 27±19 | 225±34 | 0.79±0.03 | 2.24±0.85 |
| B_15_ | 0.81±0.40 | -9.62±2.19 | 21±11 | 227±43 | 0.93±0.06 | 1.52±0.45 |
| B_40_ | 0.85±0.27 | -7.60±6.01 | 50±37 | 118±52 | 0.85±0.07 | 1.88±0.17 |
| B_80_ | 0.67±0.60 | -5.31±4.87 | 55±43 | 215±34 | 0.92±0.00 | 1.44±0.50 |
| P_5_ | 0.72±0.12 | -2.66±3.71 | 2±5 | 103±40 | 0.74±0.18 | 1.14±0.20 |
| P_15_ | 0.82±0.55 | -1.96±3.16 | 16±18 | 98±62 | 0.67±0.08 | 1.40±0.31 |
| P_40_ | 0.97±0.05 | 0.09±0.30 | 12±12 | 63±24 | 0.78±0.10 | 0.83±0.07 |
| P_80_ | 0.66±0.21 | 7.44±14.11 | 3±3 | 81±42 | 0.91±0.01 | 1.03±0.11 |
| S_5_ | 0.95±0.34 | 0.03±0.47 | 8±15 | 184±112 | 0.91±0.02 | 1.54±0.10 |
| S_15_ | 0.45±0.35 | -0.23±0.43 | 8±10 | 144±60 | 0.88±0.25 | 1.45±0.10 |
| S_40_ | 0.81±0.23 | -0.66±1.19 | 5±7 | 243±75 | 0.99±0.01 | 1.08±0.00 |
| S_80_ | 1.07±0.18 | 0.08±0.94 | 9±8 | 284±6 | 0.97±0.00 | 1.48±0.00 |

**Supplementary Table 4.** The abundances (copies/(g^-1^ dw^-1^)) of bacterial and archaeal 16S rRNA genes (16S rRNA), and nitrogen-cycling genes in study plots` soils of birch (B), pine (P) and spruce (S) stands. The first number in the sample code denotes the replicate (1-3) within a stand type and the number, given in subscript, stands for the sampling site distance (m) from the drainage ditch. *BamoA* – *amoA* of canonical ammonia-oxidizing bacteria, *AamoA* – archaeal-specific *amoA*, *CamoA* – comammox-specific *amoA,* nd *–* not detected.

| **Sample** | **Bacterial 16S rRNA** | **Archaeal 16S rRNA** | ***BamoA*** | ***AamoA*** | ***CamoA*** | ***nrfA*** | ***nifH*** | ***nirS*** | ***nirK*** | ***nosZI*** | ***nosZII*** |
| --- | --- | --- | --- | --- | --- | --- | --- | --- | --- | --- | --- |
| B1_5_ | 2.65*10^11^ | 2.64*10^9^ | 4.16*10^4^ | 6.51*10^5^ | nd | 2.07*10^6^ | 6.10*10^8^ | 4.19*10^6^ | 1.43*10^9^ | 1.24*10^7^ | 5.81*10^6^ |
| B1_15_ | 2.13*10^11^ | 5.02*10^9^ | 5.31*10^5^ | 2.99*10^6^ | nd | 1.51*10^6^ | 3.43*10^8^ | 7.07*10^5^ | 6.05*10^8^ | 1.43*10^7^ | 3.40*10^6^ |
| B1_40_ | 1.87*10^11^ | 3.62*10^9^ | 6.30*10^5^ | 2.41*10^5^ | nd | 2.23*10^5^ | 5.05*10^8^ | 3.68*10^5^ | 6.76*10^8^ | 2.52*10^7^ | 4.50*10^6^ |
| B1_80_ | 2.31*10^11^ | 6.12*10^9^ | 4.20*10^5^ | 2.03*10^5^ | nd | 8.65*10^6^ | 1.08*10^9^ | 4.27*10^5^ | 1.44*10^9^ | 4.85*10^6^ | 2.51*10^6^ |
| B2_5_ | 1.86*10^11^ | 7.55*10^8^ | 1.39*10^6^ | 1.15*10^6^ | 4.74*10^6^ | 1.46*10^6^ | 1.94*10^8^ | 1.33*10^8^ | 8.10*10^8^ | 4.95*10^7^ | 8.37*10^7^ |
| B2_15_ | 2.01*10^11^ | 1.65*10^9^ | 1.12*10^7^ | 1.02*10^7^ | 1.71*10^6^ | 1.22*10^6^ | 2.95*10^8^ | 2.03*10^8^ | 9.39*10^8^ | 1.05*10^8^ | 1.45*10^9^ |
| B2_40_ | 1.87*10^11^ | 7.50*10^8^ | 2.21*10^7^ | 2.79*10^6^ | 5.25*10^6^ | 1.56*10^6^ | 8.96*10^8^ | 3.82*10^8^ | 7.80*10^8^ | 3.61*10^8^ | 1.17*10^8^ |
| B2_80_ | 1.28*10^11^ | 1.25*10^9^ | 1.02*10^7^ | 4.69*10^6^ | 8.71*10^4^ | 2.20*10^5^ | 6.26*10^7^ | 1.75*10^8^ | 1.22*10^9^ | 4.32*10^7^ | 2.45*10^8^ |
| B3_5_ | 1.89*10^11^ | 5.43*10^8^ | 2.17*10^7^ | 1.05*10^6^ | 4.96*10^6^ | 5.93*10^5^ | 4.20*10^8^ | 2.92*10^8^ | 1.85*10^9^ | 1.38*10^8^ | 2.56*10^8^ |
| B3_15_ | 2.22*10^11^ | 1.12*10^9^ | 1.03*10^7^ | 3.39*10^6^ | 5.26*10^5^ | 5.21*10^5^ | 6.62*10^8^ | 1.37*10^8^ | 1.11*10^9^ | 1.24*10^8^ | 1.61*10^9^ |
| B3_40_ | 1.17*10^11^ | 8.19*10^8^ | 3.98*10^6^ | 3.31*10^6^ | 9.28*10^5^ | 2.20*10^5^ | 6.54*10^8^ | 1.24*10^8^ | 7.03*10^8^ | 3.12*10^8^ | 6.37*10^7^ |
| B3_80_ | 2.33*10^11^ | 3.08*10^9^ | 2.12*10^7^ | 6.58*10^6^ | 5.13*10^5^ | 2.61*10^7^ | 1.37*10^9^ | 9.45*10^7^ | 2.77*10^9^ | 6.61*10^7^ | 5.44*10^8^ |
|  |  |  |  |  |  |  |  |  |  |  |  |
| P1_5_ | 1.86*10^11^ | 4.73*10^9^ | 8.26*10^4^ | 7.66*10^4^ | nd | 2.20*10^5^ | 3.69*10^8^ | 1.28*10^5^ | 1.09*10^10^ | 6.03*10^6^ | 5.46*10^7^ |
| P1_15_ | 1.97*10^11^ | 4.25*10^9^ | 2.03*10^4^ | 5.86*10^4^ | nd | 1.22*10^5^ | 3.06*10^8^ | 7.97*10^4^ | 7.75*10^9^ | 9.86*10^6^ | 8.70*10^6^ |
| P1_40_ | 1.98*10^11^ | 4.94*10^9^ | 5.13*10^4^ | 2.61*10^4^ | nd | 5.35*10^5^ | 2.54*10^8^ | 3.60*10^5^ | 1.80*10^9^ | 6.07*10^6^ | 2.03*10^6^ |
| P1_80_ | 1.87*10^11^ | 1.25*10^10^ | 5.53*10^4^ | 3.32*10^5^ | nd | 5.73*10^5^ | 9.16*10^8^ | 1.69*10^5^ | 6.35*10^9^ | 2.30*10^7^ | 3.69*10^7^ |
| P2_5_ | 1.97*10^11^ | 5.22*10^9^ | 5.98*10^4^ | 2.44*10^6^ | nd | 5.04*10^6^ | 2.43*10^9^ | 1.62*10^6^ | 1.18*10^10^ | 5.28*10^7^ | 2.90*10^8^ |
| P2_15_ | 2.35*10^11^ | 9.77*10^9^ | 1.43*10^6^ | 7.39*10^8^ | nd | 7.36*10^5^ | 1.78*10^9^ | 1.04*10^7^ | 9.53*10^9^ | 8.54*10^7^ | 2.75*10^7^ |
| P2_40_ | 2.05*10^11^ | 9.34*10^9^ | 4.53*10^6^ | 7.39*10^7^ | nd | 4.15*10^6^ | 5.33*10^8^ | 5.65*10^6^ | 2.40*10^9^ | 2.91*10^7^ | 2.49*10^6^ |
| P2_80_ | 2.04*10^11^ | 9.36*10^9^ | 1.93*10^5^ | 6.53*10^6^ | nd | 1.04*10^6^ | 4.64*10^9^ | 6.91*10^5^ | 5.01*10^9^ | 5.44*10^7^ | 6.37*10^7^ |
| P3_5_ | 3.00*10^11^ | 8.17*10^9^ | 1.45*10^5^ | 1.46*10^6^ | nd | 4.62*10^6^ | 3.99*10^9^ | 7.85*10^5^ | 1.58*10^10^ | 6.09*10^7^ | 4.52*10^8^ |
| P3_15_ | 2.99*10^11^ | 1.97*10^10^ | 1.63*10^5^ | 1.12*10^9^ | nd | 3.71*10^5^ | 3.03*10^9^ | 1.33*10^6^ | 9.66*10^9^ | 9.46*10^7^ | 6.31*10^7^ |
| P3_40_ | 3.54*10^11^ | 7.19*10^9^ | 1.02*10^5^ | 3.41*10^7^ | nd | 3.51*10^6^ | 3.19*10^9^ | 8.89*10^5^ | 2.91*10^9^ | 4.19*10^7^ | 2.86*10^6^ |
| P3_80_ | 2.11*10^11^ | 4.41*10^9^ | 1.03*10^5^ | 4.19*10^5^ | nd | 2.35*10^5^ | 7.94*10^9^ | 1.66*10^4^ | 3.35*10^9^ | 8.30*10^7^ | 2.50*10^7^ |
|  |  |  |  |  |  |  |  |  |  |  |  |
| S1_5_ | 2.13*10^11^ | 3.48*10^9^ | 5.35*10^6^ | 6.61*10^7^ | 1.65*10^6^ | 2.59*10^6^ | 2.54*10^8^ | 2.31*10^7^ | 4.68*10^9^ | 5.76*10^7^ | 4.32*10^8^ |
| S1_15_ | 1.73*10^11^ | 2.40*10^9^ | 7.07*10^6^ | 3.10*10^7^ | 2.21*10^6^ | 1.26*10^6^ | 4.66*10^8^ | 2.25*10^8^ | 1.68*10^9^ | 1.29*10^8^ | 6.20*10^8^ |
| S1_40_ | 1.83*10^11^ | 3.61*10^9^ | 3.73*10^6^ | 5.86*10^7^ | 3.33*10^6^ | 3.68*10^5^ | 3.06*10^8^ | 1.46*10^8^ | 2.19*10^9^ | 3.16*10^8^ | 3.67*10^8^ |
| S1_80_ | 3.08*10^11^ | 4.21*10^9^ | 5.24*10^6^ | 2.68*10^8^ | 3.60*10^6^ | 1.89*10^6^ | 2.92*10^9^ | 8.16*10^7^ | 6.51*10^9^ | 8.17*10^7^ | 2.80*10^8^ |
| S2_5_ | 1.17*10^11^ | 9.90*10^8^ | 8.10*10^5^ | 1.85*10^6^ | nd | 1.24*10^6^ | 7.32*10^8^ | 4.43*10^6^ | 2.90*10^9^ | 1.40*10^7^ | 1.76*10^7^ |
| S2_15_ | 1.81*10^11^ | 2.87*10^9^ | 1.45*10^5^ | 2.40*10^4^ | nd | 8.89*10^5^ | 1.85*10^9^ | 2.64*10^5^ | 1.80*10^9^ | 2.71*10^7^ | 1.88*10^6^ |
| S2_40_ | 1.54*10^11^ | 2.26*10^9^ | 6.23*10^4^ | 1.95*10^5^ | nd | 2.89*10^5^ | 5.31*10^8^ | 8.07*10^7^ | 2.12*10^9^ | 2.46*10^8^ | 7.61*10^7^ |
| S2_80_ | 1.88*10^11^ | 8.86*10^8^ | 9.75*10^5^ | 1.63*10^6^ | 3.29*10^6^ | 9.83*10^5^ | 1.40*10^9^ | 9.97*10^7^ | 5.64*10^9^ | 1.34*10^8^ | 1.90*10^8^ |
| S3_5_ | 1.93*10^11^ | 5.15*10^9^ | 3.56*10^4^ | 4.16*10^5^ | nd | 6.56*10^6^ | 4.44*10^8^ | 2.65*10^5^ | 5.91*10^9^ | 7.92*10^6^ | 1.83*10^7^ |
| S3_15_ | 1.32*10^11^ | 8.09*10^9^ | 4.15*10^4^ | 8.76*10^3^ | nd | 7.60*10^5^ | 4.82*10^8^ | 8.22*10^5^ | 2.11*10^9^ | 1.28*10^7^ | 2.38*10^6^ |
| S3_40_ | 1.05*10^11^ | 2.35*10^9^ | 1.64*10^6^ | 7.23*10^4^ | nd | 2.44*10^5^ | 2.15*10^8^ | 2.34*10^4^ | 1.24*10^9^ | 1.66*10^7^ | 6.90*10^6^ |
| S3_5_^A^ | 1.57*10^11^ | 6.71*10^9^ | 6.73*10^4^ | 1.39*10^6^ | nd | 4.10*10^6^ | 2.65*10^8^ | 1.91*10^5^ | 4.54*10^9^ | 5.00*10^6^ | 1.93*10^7^ |
| S3_15_^A^ | 1.38*10^11^ | 8.84*10^9^ | 3.15*10^4^ | 1.35*10^7^ | nd | 9.69*10^5^ | 3.31*10^8^ | 1.02*10^5^ | 1.44*10^9^ | 1.02*10^7^ | 2.37*10^7^ |

^A^ – additional sampling sites that located at 5 and 15 m distance from the second ditch of the S3 stand

**Supplementary Table 5**. Means and standard deviations of the obtained total (good quality), bacterial (Bact) and archaeal (Arch) 16S rRNA gene sequences, as well as the samples coverage estimates (SC), number of OTUs and diversity indices (A1/S and B1/S - Inverse Simpson index, and BWPD_B_ and BWPD_A_ – balance-weighted phylogenetic diversity index for bacteria and archaea, respectively) obtained for both groups of prokaryotes per sampling plots of different forests. B – birch; P – pine; S – spruce; number in subscript shows the plots distance (m) from drainage ditch (n=3).

| Plots | No sequences | | | Coverage | | No OTUs | | Diversity indices | | | |
| --- | --- | --- | --- | --- | --- | --- | --- | --- | --- | --- | --- |
|  | Total | Bact | Arch | Bact | Arch | Bact | Arch | B1/S | A1/S | BWPD_B_ | BWPD_A_ |
| B_5_ | 59099  ±21362 | 58540  ±21198 | 203  ±82 | 0.90  ±0.02 | 0.84  ±0.13 | 3119  ±712 | 15  ±4 | 67  ±18 | 7.2  ±3.8 | 13.4  ±0.8 | 8.65  ±1.51 |
| B_15_ | 51604  ±4214 | 51042  ±4069 | 196  ±117 | 0.90  ±0.01 | 0.87  ±0.04 | 2809  ±136 | 13  ±4 | 71  ±25 | 5.8  ±0.8 | 13.3  ±0.6 | 7.47  ±1.94 |
| B_40_ | 49465  ±2492 | 48888  ±2330 | 242  ±133 | 0.89  ±0.01 | 0.82  ±0.19 | 2841  ±208 | 15  ±4 | 73  ±17 | 5.7  ±2.9 | 13.1  ±0.6 | 8.60  ±1.94 |
| B_80_ | 59065  ±7411 | 58541  ±7291 | 155  ±44 | 0.90  ±0.01 | 0.83  ±0.13 | 3027  ±196 | 13  ±3 | 78  ±15 | 5.9  ±1.2 | 13.0  ±0.6 | 5.88  ±2.1 |
| P_5_ | 57824  ±8421 | 57213  ±8305 | 121  ±6 | 0.87  ±0.02 | 0.77  ±0.05 | 3721  ±226 | 17  ±2 | 188  ±102 | 15.6  ±6.7 | 11.3  ±0.5 | 7.89  ±3.62 |
| P_15_ | 68312  ±3693 | 67708  ±3829 | 228  ±184 | 0.89  ±0.01 | 0.83  ±0.08 | 4073  ±379 | 18  ±6 | 194  ±114 | 7.4  ±2.3 | 11.1  ±0.3 | 3.18  ±2.04 |
| P_40_ | 67044  ±4398 | 66570  ±4301 | 183  ±76 | 0.89  ±0.01 | 0.68  ±0.09 | 3851  ±291 | 19  ±4 | 167  ±94 | 9.5  ±2.4 | 11.3  ±0.2 | 5.33  ±4.79 |
| P_80_ | 65472  ±13818 | 64965  ±13680 | 179  ±136 | 0.88  ±0.02 | 0.69  ±0.20 | 3842  ±468 | 17  ±5 | 134  ±53 | 14.4  ±10.3 | 11.0  ±0.1 | 4.64  ±2.23 |
| S_5_ | 36013  ±9518 | 35641  ±9314 | 161  ±183 | 0.84  ±0.06 | 0.74  ±0.21 | 2429  ±191 | 11  ±6 | 133  ±87 | 7.1  ±3.4 | 12.0  ±0.3 | 6.21  ±1.33 |
| S_15_ | 41083  ±7416 | 40567  ±7256 | 189  ±167 | 0.87  ±0.04 | 0.86  ±0.16 | 2506  ±315 | 10  ±5 | 119  ±88 | 7.6  ±2.4 | 11.7  ±0.6 | 6.29  ±2.31 |
| S_40_ | 38452  ±3194 | 37969  ±3325 | 220  ±237 | 0.85  ±0.04 | 0.79  ±0.20 | 2660  ±335 | 11  ±3 | 178  ±59 | 6.4  ±4.8 | 12.6  ±0.5 | 6.74  ±2.24 |
| **S_80_** | 33574  ±4381 | 33218  ±4213 | 161  ±169 | 0.84  ±0.03 | 0.78  ±0.23 | 2556  ±151 | 12  ±4 | 216  ±73 | 10.6  ±12.5 | 12.8  ±0.9 | 8.79  ±5.54 |

**Supplementary Table 6.** Proportions (averages and standard deviations) in the bacterial community of less abundant bacterial phylum that were significantly different between different forest groups (p<0.05 in all cases).

| Genus | Proportion (%) | | |
| --- | --- | --- | --- |
|  | Birch | Pine | Spruce |
| *Rokubacteria* | 0.29±0.07 | 0.05±0.01 | 0.23±0.13 |
| *Latescibacteria* | 0.30±0.02 | 0.05±0.02 | 0.14±0.05 |
| *Nitrospirae* | 0.30±0.30 | 0.05±0.02 | 0.13±0.08 |
| *FCPU426* | 0.07±0.01 | 0.24±0.07 | 0.15±0.06 |
| *Spirochaetes* | 0.23±0.04 | 0.08±0.02 | 0.07±0.03 |

**Supplementary Table 7.** Topological properties of the obtained prokaryotic organisms` networks of each studied forest group. Modul. – modularity, avgK - average connectivity, avgCC – average clustering coefficient, No – number of modules composed of more than five operational taxonomic units (OTUs).

| Stand | Topological properties of the obtained networks | | | | | | | | |
| --- | --- | --- | --- | --- | --- | --- | --- | --- | --- |
|  | Modul. | avgK | avgCC | Degree | Closeness | Betweenness | Eigenvector | No | OTUs |
| Birch | 0.59 | 1.5 | 0.04 | 0.04 | 0.002 | 0.02 | 0.94 | 7 | 121 |
| Pine | 0.37 | 10.7 | 0.51 | 0.18 | 0.008 | 0.04 | 0.82 | 7 | 178 |
| Spruce | 0.43 | 7.2 | 0.41 | 0.21 | 0.013 | 0.09 | 0.85 | 7 | 126 |

**Supplementary Table 8**. The proportions (%) of archaeal 16S rRNA genes in all prokaryotic 16S rRNA genes (16S) and nitrogen-cycling genes in bacterial and archaeal communities (B16S and A16S, respectively) or the prokaryotic community, as well as the ratios of functional genes in the soils of each sampling point of birch (B), pine (P) and spruce (S) stands. The first number in the sample code denotes the replicate (1-3) of a stand type and the number, given in subscript, stands for the sampling site distance (m) from the drainage ditch. *BamoA* – bacterial *amoA*, *AmoA* – archaeal *amoA*, *CamoA* – comammox-specific *amoA*, N16S – estimated abundance of *Nitrospira* 16S rRNA gene sequences, *nosZ* – total abundance of *nosZ* genes, *nir* – total abundance of *nir* genes, nd – not detected.

| **Sample** | **A16S/ 16S** | ***BamoA*/ B16S** | ***AamoA*/ A16S** | ***CamoA*/N16S** | ***nifH/ B16S*** | ***nrfA/ B16S*** | ***nirS/ B16S*** | ***nirK/ B16S*** | ***nirS/ nirK*** | ***nosZI/ B16S*** | ***nosZII/ B16S*** | ***nosZI/ nosZII*** | ***nir/nosZ*** |
| --- | --- | --- | --- | --- | --- | --- | --- | --- | --- | --- | --- | --- | --- |
| B1_5_ | 0.99 | 0.0001 | 0.02 | nd | 0.23 | 0.0008 | 0.0016 | 0.54 | 0.0029 | 0.0047 | 0.002 | 2.14 | 78.5 |
| B1_15_ | 2.31 | 0.0003 | 0.06 | nd | 0.16 | 0.0007 | 0.0003 | 0.28 | 0.0012 | 0.0067 | 0.002 | 4.20 | 34.2 |
| B1_40_ | 1.90 | 0.0003 | 0.01 | nd | 0.27 | 0.0001 | 0.0002 | 0.36 | 0.0005 | 0.0135 | 0.002 | 5.59 | 22.8 |
| B1_80_ | 2.58 | 0.0002 | 0.01 | nd | 0.47 | 0.0038 | 0.0002 | 0.63 | 0.0003 | 0.0021 | 0.001 | 1.93 | 196 |
| B2_5_ | 0.41 | 0.0008 | 0.15 | 0.43 | 0.10 | 0.0008 | 0.0715 | 0.44 | 0.1637 | 0.0267 | 0.045 | 0.59 | 7.08 |
| B2_15_ | 0.81 | 0.0056 | 0.62 | 0.16 | 0.15 | 0.0006 | 0.1010 | 0.47 | 0.2161 | 0.0522 | 0.723 | 0.07 | 0.73 |
| B2_40_ | 0.40 | 0.0118 | 0.37 | 0.59 | 0.48 | 0.0008 | 0.2039 | 0.42 | 0.4897 | 0.1925 | 0.063 | 3.08 | 2.42 |
| B2_80_ | 0.97 | 0.0080 | 0.37 | 0.01 | 0.05 | 0.0002 | 0.1368 | 0.95 | 0.1434 | 0.0338 | 0.192 | 0.18 | 4.84 |
| B3_5_ | 0.29 | 0.0115 | 0.19 | 0.76 | 0.22 | 0.0003 | 0.1547 | 0.98 | 0.1579 | 0.0729 | 0.135 | 0.54 | 5.44 |
| B3_15_ | 0.50 | 0.0046 | 0.30 | 0.12 | 0.30 | 0.0002 | 0.0617 | 0.50 | 0.1232 | 0.0560 | 0.727 | 0.08 | 0.72 |
| B3_40_ | 0.70 | 0.0034 | 0.40 | 0.38 | 0.56 | 0.0002 | 0.1057 | 0.60 | 0.1759 | 0.2663 | 0.054 | 4.89 | 2.20 |
| B3_80_ | 1.30 | 0.0091 | 0.21 | 0.11 | 0.59 | 0.0112 | 0.0405 | 1.19 | 0.0341 | 0.0283 | 0.233 | 0.12 | 4.69 |
|  |  |  |  |  |  |  |  |  |  |  |  |  |  |
| P1_5_ | 2.48 | 0.0001 | 0.01 | nd | 0.20 | 0.0001 | 0.0001 | 5.84 | 0.0001 | 0.0032 | 0.029 | 0.11 | 179 |
| P1_15_ | 2.11 | 0.0001 | 0.01 | nd | 0.16 | 0.0001 | 0.0001 | 3.93 | 0.0001 | 0.0050 | 0.004 | 1.13 | 417 |
| P1_40_ | 2.44 | 0.0001 | 0.01 | nd | 0.13 | 0.0003 | 0.0002 | 0.91 | 0.0002 | 0.0031 | 0.001 | 2.99 | 222 |
| P1_80_ | 6.27 | 0.0001 | 0.01 | nd | 0.49 | 0.0003 | 0.0001 | 3.41 | 0.0001 | 0.0123 | 0.020 | 0.62 | 106 |
| P2_5_ | 2.58 | 0.0001 | 0.05 | nd | 1.23 | 0.0026 | 0.0008 | 5.99 | 0.0001 | 0.0268 | 0.147 | 0.18 | 34.4 |
| P2_15_ | 3.99 | 0.0006 | 7.56 | nd | 0.76 | 0.0003 | 0.0044 | 4.05 | 0.0011 | 0.0363 | 0.012 | 3.10 | 84.5 |
| P2_40_ | 4.35 | 0.0022 | 0.79 | nd | 0.26 | 0.0020 | 0.0028 | 1.17 | 0.0024 | 0.0142 | 0.001 | 11.7 | 76.3 |
| P2_80_ | 4.38 | 0.0001 | 0.07 | nd | 2.27 | 0.0005 | 0.0003 | 2.45 | 0.0001 | 0.0266 | 0.031 | 0.85 | 42.5 |
| P3_5_ | 2.65 | 0.0001 | 0.02 | nd | 1.33 | 0.0015 | 0.0003 | 5.28 | 0.0001 | 0.0203 | 0.151 | 0.13 | 30.9 |
| P3_15_ | 6.18 | 0.0001 | 5.67 | nd | 1.01 | 0.0001 | 0.0004 | 3.23 | 0.0001 | 0.0316 | 0.021 | 1.50 | 61.2 |
| P3_40_ | 1.99 | 0.0001 | 0.47 | nd | 0.90 | 0.0010 | 0.0003 | 0.82 | 0.0003 | 0.0118 | 0.001 | 14.7 | 65.0 |
| P3_80_ | 2.05 | 0.0001 | 0.01 | nd | 3.77 | 0.0001 | 0.0001 | 1.59 | 0.0001 | 0.0394 | 0.012 | 3.32 | 31.0 |
|  |  |  |  |  |  |  |  |  |  |  |  |  |  |
| S1_5_ | 1.61 | 0.0025 | 1.90 | 0.44 | 0.12 | 0.0012 | 0.0108 | 2.20 | 0.0049 | 0.0271 | 0.203 | 0.13 | 9.61 |
| S1_15_ | 1.37 | 0.0041 | 1.29 | 0.39 | 0.27 | 0.0007 | 0.1301 | 0.97 | 0.1338 | 0.0745 | 0.359 | 0.21 | 2.54 |
| S1_40_ | 1.94 | 0.0026 | 1.62 | 0.96 | 0.17 | 0.0002 | 0.0797 | 1.20 | 0.0665 | 0.1730 | 0.201 | 0.86 | 3.43 |
| S1_80_ | 1.35 | 0.0017 | 6.37 | 1.22 | 0.95 | 0.0006 | 0.0265 | 2.11 | 0.0125 | 0.0265 | 0.091 | 0.29 | 18.2 |
| S2_5_ | 0.84 | 0.0007 | 0.19 | nd | 0.63 | 0.0011 | 0.0038 | 2.48 | 0.0015 | 0.0120 | 0.015 | 0.79 | 91.7 |
| S2_15_ | 1.56 | 0.0001 | 0.01 | nd | 1.02 | 0.0005 | 0.0001 | 0.99 | 0.0001 | 0.0150 | 0.001 | 14.4 | 62.1 |
| S2_40_ | 1.45 | 0.0001 | 0.01 | nd | 0.35 | 0.0002 | 0.0526 | 1.38 | 0.0381 | 0.1605 | 0.050 | 3.24 | 6.82 |
| S2_80_ | 0.47 | 0.0005 | 0.18 | 0.60 | 0.74 | 0.0005 | 0.0530 | 3.00 | 0.0177 | 0.0711 | 0.101 | 0.70 | 17.7 |
| S3_5_ | 2.60 | 0.0001 | 0.01 | nd | 0.23 | 0.0034 | 0.0002 | 3.06 | 0.0001 | 0.0041 | 0.009 | 0.43 | 225 |
| S3_15_ | 5.76 | 0.0001 | 0.01 | nd | 0.36 | 0.0006 | 0.0006 | 1.60 | 0.0004 | 0.0096 | 0.002 | 5.36 | 140 |
| S3_40_ | 2.19 | 0.0016 | 0.01 | nd | 0.20 | 0.0002 | 0.0001 | 1.18 | 0.0001 | 0.0158 | 0.007 | 2.41 | 52.9 |
| S3_5_^A^ | 4.10 | 0.0001 | 0.02 | nd | 0.17 | 0.0026 | 0.0001 | 2.89 | 0.0001 | 0.0032 | 0.012 | 0.26 | 187 |
| S3_15_^A^ | 6.04 | 0.0001 | 0.15 | nd | 0.24 | 0.0007 | 0.0001 | 1.05 | 0.0001 | 0.0074 | 0.017 | 0.43 | 42.5 |

^A^- additional sampling sites that located at 5 and 15 m distance from the second ditch of the S3 stand

**Supplementary Table 9**. Results of the distance-based redundancy analysis with soil chemical variables and root parameters as predictors. The analysis was performed based on the Bray-Curtis distance matrices of the whole prokaryotic community and microbial ecological network modules. TN – total nitrogen, DN - dissolved nitrogen, TP – total phosphorous, DOC – dissolved organic carbon, FRBt – fine root biomass of trees.

| Community type | Soil chemical variables | | Root traits | |
| --- | --- | --- | --- | --- |
|  |  |  |  |  |
|  |  |  |  |  |
|  | Variation explained (%) | Variables in the model | Variation explained (%) | Parameter in the model |
| All forests | | | | |
|  | 77.5 | pH, NH_4_, NO_3_, K, Ca, TC, PO_4_ | 14.6 | FRB_t_ |
| Birch forests | | | | |
| Whole community | 57.5 | NH_4_, K | - |  |
| BM1 | 40.4 | K | - |  |
| BM2 | - |  | - |  |
| BM3 | 43.6 | K | - |  |
| BM4 | - |  | - |  |
| BM5 | - |  | - |  |
| BM6 | 42.5 | K | - |  |
| BM7 | - |  | - |  |
| Pine forests | | | | |
| Whole community | 71.0 | C/N, pH | 35.0 | FRB_t_ |
| PM1 | 55.3 | DN | - |  |
| PM2 | 77.8 | C/N, S | 35.7 | FRB_t_ |
| PM3 | 37.5 | NH_4_ | - |  |
| PM4 | 76.7 | K, TP | 33.2 | FRB_t_ |
| PM5 | 89.8 | S, K, C/N | 46.1 | FRB_t_ |
| PM6 | 77.0 | pH, DN | 45.3 | FRB_t_ |
| PM7 | 47.3 | C/N | 58.1 | FRB_t_ |
| Spruce forests | | | | |
| Whole community | 92.2 | pH, TN, TP, Ca | 25.7 | FRB_t_ |
| SM1 | 85.9 | pH, N, NO_3_, DOC | 30.7 | FRB_t_ |
| SM2 | 88.8 | pH, Ca | 41.4 | FRB_t_ |
| SM3 | 78.8 | pH | - |  |
| SM4 | 93.0 | pH, N, NO_3_, NH_4_ | 29.7 | FRB_t_ |
| SM5 | 71.3 | pH, TC, NO_3_ | - |  |
| SM6 | 83.4 | TN, DOC, NH_4_ | - |  |
| SM7 | 78.3 | DOC, Ca | 31.7 | FRB_t_ |

– no variation was explained by these parameters.

**Supplementary Table 10.** The proportions of the nitrifying archaeal and bacterial genera (means and standard deviations, %, n=3) in communities of the respective prokaryotic group of the studied plots` soils. B – birch forests, P – pine forests, S – spruce forests, AOB – ammonia-oxidizing bacteria, NOB – nitrite-oxidizing bacteria, AOA – ammonia-oxidizing archaea. The numbers at the subscripts of the plots abbreviations stand for the plots distance (m) from the drainage ditch.

| Group | Genus | Plots | | | | | | | | | | | |
| --- | --- | --- | --- | --- | --- | --- | --- | --- | --- | --- | --- | --- | --- |
|  |  | P_5_ | P_15_ | P_40_ | P_80_ | B_5_ | B_15_ | B_40_ | B_80_ | S_5_ | S_15_ | S_40_ | S_80_ |
| AOB | *Nitrosomonas* | 0.009  ±0.006 | 0.002  ±0.003 | 0.003  ±0.003 | 0.006  ±0.006 | 0 | 0.011  ±0.018 | 0 | 0.001  ±0.002 | 0 | 0.001  ±0.003 | 0.014  ±0.024 | 0 |
|  | *Nitrosospira* | 0.012  ±0.004 | 0.004  ±0.004 | 0.013  ±0.012 | 0.010  ±0.013 | 0.002  ±0.004 | 0.002  ±0.003 | 0 | 0.004  ±0.006 | 0 | 0.006  ±0.008 | 0.002  ±0.004 | 0 |
| NOB | *Nitrobacter* | 0.823  ±0.430 | 0.822  ±0.484 | 0.779  ±0.418 | 0.761  ±0.236 | 0.521  ±0.160 | 0.616  ±0.076 | 0.448  ±0.103 | 0.466  ±0.038 | 0.578  ±0.207 | 0.514  ±0.218 | 0.832  ±0.203 | 1.050  ±0.281 |
|  | *Nitrospira* | 0.347  ±0.250 | 0.262  ±0.246 | 0.262  ±0.190 | 0.278  ±0.260 | 0.057  ±0.039 | 0.044  ±0.007 | 0.046  ±0.006 | 0.020  ±0.006 | 0.078  ±0.069 | 0.110  ±0.147 | 0.096  ±0.082 | 0.194  ±0.139 |
|  | *Nitrospina* | 0 | 0 | 0.001  ±0.002 | 0.001  ±0.002 | 0 | 0 | 0 | 0.003  ±0.002 | 0.003  ±0.006 | 0.001  ±0.000 | 0.002  ±0.004 | 0.004  ±0.006 |
| AOA | *Ca Nitrosotalea* | 2.3  ±0.1 | 1.3  ±1.3 | 0.5  ±0.9 | 1.5  ±1.9 | 13.6  ±15.4 | 20.2  ±17.6 | 12.3  ±13.6 | 0 | 11.4  ±22.7 | 6.0  ±8.0 | 3.3  ±5.8 | 3.8  ±3.0 |
|  | *Ca Nitrocosmicus* | 3.9  ±3.6 | 5.9  ±1.6 | 0 | 0.8  ±1.4 | 1.1  ±2.0 | 0.8  ±1.3 | 0.9  ±1.0 | 0.9  ±1.5 | 2.6  ±4.4 | 0.6  ±1.3 | 1.1  ±1.9 | 0 |
|  | *Ca Nitrososphaera* | 1.5  ±1.3 | 0 | 0 | 0 | 0 | 0 | 0 | 0 | 0.2  ±0.4 | 1.8  ±3.6 | 0 | 0 |
|  | *Ca Nitrosopelagicus* | 0 | 0 | 1.0  ±1.7 | 0 | 1.1  ±2.0 | 0 | 0 | 0 | 0 | 0 | 1.1  ±1.9 | 0 |
|  | *Ca Nitrosopumius* | 2.5  ±2.4 | 0.2  ±0.4 | 7.4  ±8.8 | 4.7  ±5.3 | 1.5  ±1.8 | 1.4  ±2.4 | 1.4  ±2.5 | 2.9  ±0.6 | 0 | 0 | 0.7  ±1.2 | 0 |
|  | *Ca Nitrosotenuis* | 3.8  ±3.4 | 2.4  ±0.3 | 3.2  ±2.8 | 2.0  ±2.7 | 0.4  ±0.7 | 5.8  ±10.1 | 0.3  ±0.5 | 0.8  ±1.4 | 1.1  ±1.7 | 0 | 3.8  ±3.3 | 2.9  ±4.2 |

**Supplementary Table 11.** Significant Pearson correlations between the microbiological parameters and gas emissions from the whole soil column measured *in situ* at the soil sampling day and from the 0-10 cm soil layer measured in the laboratory as well as the sink (N_2_0_10cm_/ (N_2_0+N_2_)_10cm_) for across all the studied forests (All, n= 37) and for the birch (B), pine (P) and spruce (S) forests separately (n= 12). 1/S_B_ – Inverse Simpson Index for the bacterial community, BWPD_B_ and BWPD_A_ – balance-weighted phylogenetic diversity index for bacterial and archaeal communities, respectively; B16S - bacterial 16S rRNA gene abundance; A16S – archaeal 16S rRNA gene abundance; 16S – total prokaryotic 16S rRNA gene abundance; *BamoA* – abundance of bacterial *amoA* gene; *AamoA* – abundance of archaeal *amoA* gene; *amoA* – abundance of total *amoA* genes; *nir* – abundance of total *nir* genes; *nosZ* – abundance of total *nosZ* genes.

| **Gene parameter** | ***In situ* gas emission** | | | | **Emissions from 0-10 cm soil layer** | | | | | | | | |
| --- | --- | --- | --- | --- | --- | --- | --- | --- | --- | --- | --- | --- | --- |
|  | **N_2_O** | | | | **N_2_O** | | | **N_2_** | | | | **Sink** | |
|  | **All** | **B** | **P** | **S** | **All** | **B** | **P** | **All** | **B** | **P** | **S** | **All** | **P** |
| **1/S_B_** |  |  | -0.69  * | 0.65  * | 0.46  ** |  |  |  |  |  |  | 0.50  ** |  |
| **BWPD_B_** | -0.47  ** |  |  |  | 0.53  ** |  |  | -0.34  * |  |  |  | 0.62  *** |  |
| **BWPD_A_** | -0.34  * |  |  |  | 0.37  * |  |  |  |  |  |  | 0.45  ** | 0.72  ** |
| **B16S** |  |  |  | 0.62  * |  |  |  |  |  | 0.77  * |  |  |  |
| **A16S** | -0.60  *** |  |  |  |  |  |  |  |  |  |  | 0.40  * |  |
| ***nifH*** |  |  |  |  | 0.38  * |  | 0.62  * |  |  |  |  | 0.35  * | 0.61  * |
| ***nifH/B16S*** |  |  |  |  |  |  | 0.82  ** |  | 0.67  * |  |  |  | 0.80  ** |
| ***BamoA*** | 0.53  ** |  | -0.78  ** | 0.68  * |  |  |  | 0.44  ** |  |  |  | -0.42  ** |  |
| ***BamoA*/16S** |  |  |  | 0.80  ** |  |  |  | 0.40  * |  |  |  |  |  |
| **A*amoA*** |  |  | -0.81  ** | 0.69  * |  |  |  |  |  | 0.65  * |  |  |  |
| ***AamoA*/16S** |  |  | -0.60  * | 0.67  * |  |  |  |  |  |  |  |  |  |
| ***amoA*** |  |  |  | 0.65  * |  |  |  |  |  |  |  |  |  |
| ***amoA/16S*** |  |  | -0.60  * | 0.69  * |  |  |  |  |  |  |  |  |  |
| ***CamoA*** | 0.72  *** | 0.74  ** |  | 0.65  * |  |  |  | 0.54  *** |  |  |  |  |  |
| ***CamoA/B16S*** | 0.71  *** | 0.75  ** |  | 0.58  * |  |  |  | 0.53  *** |  |  |  |  |  |
| ***CamoA/BamoA*** | 0.68  *** | 0.67  * |  | 0.56  * |  |  |  | 0.51  ** |  |  |  |  |  |
| ***CamoA/amoA*** | 0.69  *** | 0.82  ** |  |  |  |  |  | 0.55  *** |  |  |  | -0.34  * |  |
| ***nirS*** | 0.63  *** |  |  | 0.71  ** | 0.38  * |  |  |  |  |  |  | -0.37  * |  |
| ***nirS/B16S*** | 0.43  * |  | -0.68  * |  |  |  |  |  |  |  |  |  |  |
| ***nirK*** | -0.38  * |  |  |  | 0.46  ** |  |  |  |  |  |  | 0.53  ** |  |
| ***nirK/B16S*** |  |  |  |  | 0.66  *** | 0.64  * |  |  | 0.58  * |  |  | 0.68  *** |  |
| ***nirS/nirK*** | 0.42  * |  | -0.60  * | -0.60  * | -0.43  * |  |  |  |  |  |  | -0.47  * |  |
| **nosZI** | 0.36  * |  | -0.73  ** | 0.60  * |  |  |  |  |  |  |  |  |  |
| ***nosZI/nosZ*** |  |  | -0.65  * |  |  |  |  |  |  |  |  |  |  |
| ***nosZI/B16S*** |  |  | -0.72  ** |  |  |  | 0.66  * |  |  |  |  |  | 0.66  * |
| **nosZII** | 0.49  ** |  |  | 0.72  ** |  |  |  |  |  |  |  |  |  |
| ***nosZII/B16S*** | -0.42  * |  |  | 0.70  ** |  |  |  |  |  |  |  |  |  |
| ***nosZII/nosZ*** |  |  |  |  |  |  |  |  |  |  | 0.58* |  |  |
| ***nosZI/ nosZII*** |  |  |  |  |  |  |  |  |  | 0.70  * |  |  |  |
| ***nosZ*** |  |  |  | 0.76  ** |  |  |  |  |  |  |  |  |  |
| ***nir/nosZ*** |  |  |  | -0.59  ** | 0.51  ** |  | -0.61  * |  |  |  |  | 0.57  ** | -0.62  * |
| ***nrfA*** |  |  |  |  |  |  |  |  |  |  | 0.60* |  |  |
| ***nrfA/B16S*** |  |  |  |  |  |  |  | 0.62  ** |  |  |  |  |  |
| ***nrfA/16S*** |  |  |  |  |  |  |  |  | 0.62  * |  |  |  |  |

*p<0.05; **p<0.01

**Supplementary Table 12.** Bacterial and archaeal genera that were on top of the most significant organisms related (according to the RFR) to the abundances of N-cycling genes and/or the N gas parameters and that proportions were significantly different (based on Kruskal-Wallis rank sum test) between the forest groups. The related gas parameters were: N_2_O emission from 0-10 cm soil layer (N_2_O_10_) and sink across all the studied forests and N_2_O emission from the whole soil column in the pine forests (N_2_O^P^). The genera found in the ecological network (Fig. S2) and related to the gas parameters are marked with the numbers (1 - N_2_O_10_, 2 - sink, and 3 - N_2_O^P^) at the superscript of the genera names. B - birch forests; S – spruce forests; P - pine forest.

| Genus | Phylum/class | B&P | B&S | P&S | Gene/gas parameter |
| --- | --- | --- | --- | --- | --- |
| 0319-6G20 | *Deltaproteobacteria* | B<P* | B<S* | ns | *nifH^+^* |
| *Aquisphaera*^1^ | *Planctomycetes* | B<P*** | ns | S<P*** | *nifH^+^, nosZI*^-^ |
| *Bathyarchaeia* | *Crenarchaeota* | ns | S<B** | ns | *nifH ^-^, nirK*^-^ |
| *Bryobacter*^2^ | *Acidobacteria* | B<P*** | B<S* | S<P* | *nifH^+^* |
| *Ca. Koribacter*^1,3^ | *Acidobacteria* | B<P** | ns | S<P** | *nifH^+^* |
| *Ca._Solibacter*^1,3^ | *Acidobacteria* | B<P* | ns | ns | *nifH^+^, nrfA^+^* |
| *Methanomassiliicoccus* | *Euryarchaeota* | ns | S<B* | ns | *nifH^-^* |
| *Occallatibacter*^1^ | *Acidobacteria* | B<P*** | B<S* | S<P** | *niH^+^, nirK^+^*, nosZI^+^ |
| *Pajaroellobacter*^1,3^ | *Deltaproteobacteria* | B<P** | B<S* | ns | *nifH^+^* |
| *Ferruginibacter* | *Bacteroidetes* | P<B*** | ns | P<S** | *nrfA^+^* |
| A21b^1^ | *Gammaproteobacteria* | P<B*** | ns | P<S* | *BamoA^+^, nirS^+^* |
| Amb-16S-1323 | *Alphaproteobacteria* | P<B*** | S<B* | P<S* | *BamoA^+^* |
| JG30-KF-AS9 | *Chloroflexi* | P<B*** | ns | ns | *BamoA^+^*, *nosZII^+^* |
| *Pseudolabrys*^1,3^ | *Alphaproteobacteria* | P<B** | S<B* | ns | *BamoA^+^*, *nirS^+^,* *nosZII^+^* |
| *Ca. Nitrosopumilus* | *Thaumarchaeota* | ns | S<B** | S<P* | *AamoA^+^* |
| *Ca. Udaeobacter*^2^ | *Verrucomicrobia* | P<B*** | ns | P<S** | *nirS^+^* |
| *Gemmatimonas*^3^ | *Gemmatimonadetes* | P<B*** | S<B* | P<S* | *nirS^+^* |
| KF-JG30-B3 | *Alphaproteobacteria* | P<B*** | ns | ns | *nirS^+^* |
| mle1-7 | *Gammaproteobacteria* | P<B*** | ns | ns | *nirS^+^* |
| 37-13^1,2^ | *Bacteroidetes* | B<P*** | B<S* | S<P** | *nirK^+^* |
| CWT_CU03-E12^1,3^ | *Bacteroidetes* | P<B*** | S<B** | ns | *nirK^+^* |
| *Rhodanobacter* | *Gammaproteobacteria* | P<B*** | S<B* | P<S** | *nirK*^-^ |
| SM1A02 | *Planctomycetes* | P<B*** | ns | ns | *nirK*^-^ |
| SM2D12^1,3^ | *Alphaproteobacteria* | ns | B<S* | ns | *nirK^+^* |
| WD2101_soil_group^1^ | *Planctomycetes* | B<P*** | B<S* | ns | *nirK^+^* |
| GOUTA6^3^ | *Gammaproteobacteria* | P<B*** | ns | ns | *nosZI*^-^ |
| *Haliangium*^3^ | *Deltaproteobacteria* | P<B** | ns | ns | *nosZI^-^, nosZII^+^* |
| OLB12 | *Bacteroidetes* | P<B* | ns | ns | *nosZII^+^* |
| *Cycloclasticus* | *Gammaproteobacteria* | P<B* | S<B** | ns | N_2_O^P^*^+^* |
| *Terrimonas* | *Bacteroidetes* | P<B*** | ns | P<S** | N_2_O^P^*^+^* |
| *Edaphobacter* | *Acidobacteria* | ns | S<B* | ns | N_2_O_10_*^-^* |
| SB-5 | *Bacteroidetes* | P<B*** | S<B*** | ns | N_2_O_10_*^+^*, sink^-^ |
| *Thiobacillus* | *Gammaproteobacteria* | P<B*** | S<B*** | ns | N_2_O_10_*^+^*, sink^-^ |
| vadinHA17 | *Bacteroidetes* | P<B*** | S<B*** | ns | N_2_O_10_*^+^*, sink^-^ |
| *Anaerolinea* | *Chloroflexi* | P<B*** | S<B*** | ns | sink^-^ |

*p<0.05; **p<0.01; ***p<0.001; ^+^ positive correlation; ^-^ negative correlation

# Supplementary References

Butterbach-Bahl, K., Willibald, G., Papen, H. (2002). Soil core method for direct simultaneous determination of N2 and N2O-emissions from forest soil. Plant Soil 240, 105–116. Doi: 10.1023/A:1015870518723

Dethlefsen, L., Huse, S., Sogin, M.L., Relman, D.A. (2008). The pervasive effects of an antibiotic on the human gut microbiota, as revealed by deep 16s rRNA sequencing. PLoS Biology, 6(11), 2383–2400. https://doi.org/10.1371/journal.pbio.0060280

Espenberg, M., Truu, M., Mander, Ü., Kasak, K., Nõlvak, H., Ligi, T., et al. (2018). Differences in microbial community structure and nitrogen cycling in natural and drained tropical peatland soils. Sci. Rep. 8(1), 4742. https://doi.org/10.1038/s41598-018-23032-y

Henry, S., Baudoin, E., López-Gutiérrez, J.C., Martin-Laurent, F., Brauman, A., Philippot, L. (2004). Quantification of denitrifying bacteria in soils by nirK gene targeted real-time PCR. J. Microbiol. Meth. 59(3), 327–335. https://doi.org/10.1016/j.mimet.2004.07.002

Henry, S., Bru, D., Stres, B., Hallet, S., Philippot, L. (2006). Quantitative detection of the nosZ gene, encoding nitrous oxide reductase, and comparison of the abundances of 16S rRNA, narG, nirK, and nosZ genes in soils. Appl. Environ. Microbiol. 72(8), 5181–5189. https://doi.org/10.1128/AEM.00231-06

Hirano, Y., Noguchi, K., Ohashi, M., Hishi, T., Makita, N., Fujii, S., et al. (2009). A new method for placing and lifting root meshes for estimating fine root production in forest ecosystems. Plant Root 3, 26–31. Doi: 10.3117/plantroot.3.26

Jones, C.M., Graf, D.R.H., Bru, D., Philippot, L., Hallin, S. (2013). The unaccounted yet abundant nitrous oxide-reducing microbial community: a potential nitrous oxide sink. ISME J. 7(2), 417–426. https://doi.org/10.1038/ismej.2012.125

Kandeler, E., Deiglmayr, K., Tscherko, D., Bru, D., Philippot, L. (2006). Abundance of narG, nirS, nirK, and nosZ genes of denitrifying bacteria during primary successions of a glacier foreland. Appl. Environ. Microbiol. 72(9), 5957–5962. https://doi.org/10.1128/AEM.00439-06

Kriiska, K., Frey, J., Asi, E., Kabral, N., Uri, V., Aosaar, J., et al. (2019). Variation in annual carbon fluxes affecting the SOC pool in hemiboreal coniferous forests in Estonia. Forest Ecol. Manag. 433, 419−430. Doi: 10.1016/j.foreco.2018.11.026.

Liu, Z., Lozupone, C., Hamady, M., Bushman, F.D., Knight, R. (2007). Short pyrosequencing reads suffice for accurate microbial community analysis. Nucleic Acids Res. 35**:**e120. doi: 10.1093/nar/gkm541

Loftfield, N., Flessa, H., Augustin, J., Beese, F. (1997). Automated gas chromatographic system for rapid analysis of the atmospheric trace gases methane, carbon dioxide, and nitrous oxide. J. Environ. Qual. 26, 560–564. Doi: 10.2134/jeq1997.00472425002600020030x

Pruesse, E., Quast, C., Knittel, K., Fuchs, B.M., Ludwig, W., Peplies, J., et al. (2007). SILVA: A comprehensive online resource for quality checked and aligned ribosomal RNA sequence data compatible with ARB. Nucleic Acids Res. 35(21), 7188–7196. https://doi.org/10.1093/nar/gkm864

Rognes, T., Flouri, T., Nichols, B., Quince, C., Mahé, F. (2016). VSEARCH: A versatile open source tool for metagenomics. PeerJ, 2016(10). https://doi.org/10.7717/peerj.2584

Rotthauwe, J.H., Witzel, K.P., Liesack, W. (1997). The ammonia monooxygenase structural gene amoa as a functional marker: Molecular fine-scale analysis of natural ammonia-oxidizing populations. Appl. Environ. Microbiol. 63(12), 4704–4712. https://doi.org/10.1128/aem.63.12.4704-4712.1997

Ruijter, J.M., Ramakers, C., Hoogaars, W.M.H., Karlen, Y., Bakker, O., van den Hoff M.J.B., et al. (2009). Amplification efficiency: Linking baseline and bias in the analysis of quantitative PCR data. Nucleic Acids Res. 37, e45. Doi:10.1093/nar/gkp045

Takeuchi, J. (2006). Habitat segregation of a functional gene encoding nitrate ammonification in estuarine sediments. Geomicrobiol. J. 23:75–87. https://doi.org/10.1080/01490450500533866

Tourna, M., Freitag, T.E., Nicol, G.W., Prosser, J.I. (2008). Growth, activity and temperature responses of ammonia-oxidizing archaea and bacteria in soil microcosms. Environ. Microbiol. 10(5), 1357–1364. https://doi.org/10.1111/j.1462-2920.2007.01563.x

Schloss, P.D., Westcott, S.L., Ryabin, T., Hall, J.R., Hartmann, M., Hollister, E.B., et al. (2009). Introducing mothur: Open-Source, Platform-Independent, Community-Supported Software for Describing and Comparing Microbial Communities. Appl. Environ. Microbiol. 75(23), 7537–7541. https://doi.org/10.1128/AEM.01541-09

Swerts, M., Uytterhoeven, G., Merckx, R., Vlassek, K. (1995). Semicontinuous measurement of soil atmosphere gases with gas-flow soil core method. Soil Sci. Soc. Am. J. 59, 1336–1342. Doi: 10.2136/sssaj1995.03615995005900050020x

Ueda, T., Suga, Y., Yahiro, N., Matsuguchi, T. (1995). Remarkable N2-fixing bacterial diversity detected in rice roots by molecular evolutionary analysis of nifH gene sequences. J. Bacteriol. 177(5), 1414–1417. https://doi.org/10.1128/jb.177.5.1414-1417.1995

Wang, M., Huang, G., Zhao, Z., Dang, C., Liu, W., Zheng, M. (2018). Newly designed primer pair revealed dominant and diverse comammox amoA gene in full-scale wastewater treatment plants. Bioresource Technol. 270, 580–587. https://doi.org/10.1016/j.biortech.2018.09.089

Wei, W., Isobe, K., Nishizawa, T., Zhu, L., Shiratori, Y., Ohte, N., et al. (2015). Higher diversity and abundance of denitrifying microorganisms in environments than considered previously. ISME J. 9(9), 1954–1965. https://doi.org/10.1038/ismej.2015.9

Westcott, S. L., and Schloss, P. D. (2015). De novo clustering methods outperform reference-based methods for assigning 16S rRNA gene sequences to operational taxonomic units. PeerJ, 2015(12), e1487. https://doi.org/10.7717/peerj.1487

Zhang, J., Kobert, K., Flouri, T., Stamatakis, A. (2014). PEAR: A fast and accurate Illumina Paired-End reAd mergeR. Bioinformatics, 30(5), 614–620. https://doi.org/10.1093/bioinformatics/btt593
